# Humic Acid-Stabilized Biogenic FeS Nanoparticles for Cr(VI) Removal Under Simulated Acidic Mine Drainage Conditions: Optimization and Interfacial Transformation Pathways

**DOI:** 10.3390/molecules31060962

**Published:** 2026-03-12

**Authors:** Mengjia Dai, Junzhen Di, Min Zhang

**Affiliations:** 1 College of Mining, Liaoning Technical University, Fuxin 123000, China; 18342812950@163.com; 2 College of Civil Engineering, Liaoning Technical University, Fuxin 123000, China; 3 College of Environmental Science and Engineering, Liaoning Technical University, Fuxin 123000, China; 19824856906@163.com

**Keywords:** nano-FeS, SRB, HA, AMD, Cr(VI) removal

## Abstract

Acidic mine drainage (AMD) poses a severe global environmental threat due to its high acidity and elevated levels of toxic hexavalent chromium (Cr(VI)), for which biogenic iron sulfide (FeS) nanoparticles have emerged as a promising remediation agent; however, their practical application is hindered by aggregation and oxidative deactivation. This research synthesized biogenic FeS nanoparticles via sulfate-reducing bacteria (SRB) and employed humic acid (HA) as a stabilizing agent to enhance Cr(VI) removal performance in simulated AMD conditions. Single-factor experiments combined with response surface methodology identified the optimal biosynthetic conditions for FeS: yeast extract powder dosage of 2.2 g/L, Fe/S molar ratio of 0.8, and NH_4_Cl dosage of 3.1 g/L. Under these conditions, the material achieved 84.25% Cr(VI) removal, with the Fe/S molar ratio identified as the most influential parameter governing synthesis and performance. Introducing HA at an optimal dosage of 2 mg/L drove marked improvements in both nanoparticle yield and reactivity: FeS yield increased to 1096.26 mg/L, Cr(VI) removal efficiency reached 99.62%, and residual Cr(VI) dropped from 15.75 mg/L to just 0.38 mg/L. Kinetic and isotherm analyses, paired with SEM/TEM imaging and zeta potential measurements, revealed that HA stabilization improved particle dispersion and reduced lamellar stacking, resulting in a surface-controlled Cr(VI) removal process. FTIR and 2D-COS analyses demonstrated that HA-derived oxygen-containing functional groups, including O–H/N–H, C=O, and C–O moieties, played a central role in interfacial interactions during Cr(VI) sequestration. XRD results confirmed that Cr(VI) was reduced to Cr(III) and primarily immobilized as low-solubility CrOOH and Cr_2_S_3_, while the formation of Fe–Cr spinel-like phases remains tentative without X-ray Photoelectron Spectroscopy (XPS) validation. Further investigation via surface-sensitive spectroscopy and dynamic leaching tests is needed to fully assess the long-term stability of the reaction products.

## 1. Introduction

Acid mine drainage (AMD), an inevitable byproduct of global mining operations, is defined by its extreme acidity, high sulfate loads, and elevated concentrations of potentially toxic elements (PTEs), including Cr, Co, Ni, Cu, Zn, As, Mo, Sb, Pb, and Cd [1,2]. These contaminants spread widely through rainfall runoff and subsurface seepage, and their non-biodegradable nature means they persist and accumulate in the environment, inflicting lasting damage on adjacent ecosystems. The ecological harm of AMD is multifaceted: low pH directly damages the cell membranes of aquatic organisms, inhibits critical enzyme activity, and can drive local population extinctions, while heavy metals bioaccumulate through the food web, ultimately posing significant risks to human health [3]. For instance, chronic exposure to Cr(VI)-contaminated water is linked to severe health outcomes, including skin ulceration, DNA damage, and lung cancer, making AMD pollution in mining areas an urgent environmental priority worldwide.

Of these PTEs, Cr(VI) stands out as one of the most hazardous. Its high mobility and strong oxidizing capacity allow it to spread rapidly in aquatic environments, where it typically exists as highly soluble CrO_4_^2−^, HCrO_4_^−^, and Cr_2_O_7_^2−^ species, amplifying its ability to move through ecosystems and enter the food chain [1,2]. The scale of this pollution is alarming: Cr(VI) concentrations in European mining waters routinely exceed regulatory standards by 10 to 100 times, with levels reaching 120 mg/L in a lead–zinc mine in southern China, and 56 mg/L detected in the AMD of an abandoned coal mine [4,5,6,7]. Even in sediments from the Dabaoshan mining area in Guangdong Province, Cr(VI) levels as high as 147.86 mg/kg have been recorded—far exceeding the 0.05 mg/L limit set for surface water in China (GB 3838-2002) [3,7]. Addressing Cr(VI) contamination in AMD is therefore an urgent environmental priority worldwide.

Despite the urgency of this issue, conventional remediation strategies for Cr(VI) are plagued by critical limitations. Chemical precipitation, the most widely used method, requires large inputs of lime and other neutralizing agents, and generates vast volumes of heavy metal-laden sludge that carries a high risk of secondary pollution [5,8]. Ion exchange resins offer high removal efficiency but are prohibitively expensive for large-scale mining applications, prone to rapid saturation, and require complex regeneration processes [5]. Adsorbents such as activated carbon, meanwhile, suffer from limited adsorption capacity, particularly for high-concentration Cr(VI) wastewater under acidic conditions [8].

In recent years, nano-FeS has emerged as a highly promising alternative for Cr(VI) remediation, thanks to its exceptional specific surface area, strong reducing power, and inherent environmental compatibility [9]. Unlike conventional iron-based materials such as nano-zero-valent iron (nZVI), nano-FeS features a sulfide shell that effectively shields the internal reactive sites from oxidative loss, accelerates electron transfer, and delivers superior Cr(VI) removal performance [9]. For instance, sulfurized nZVI (S-NZVI) with a S/Fe molar ratio of 0.5 achieved 98% Cr(VI) removal at pH 2, vastly outperforming its unsulfurized counterpart [9]. What makes nano-FeS particularly effective is its synergistic removal mechanism: it first adsorbs Cr(VI) to its surface via electrostatic attraction, then uses structural Fe(II) and sulfide to reduce Cr(VI) to far less toxic Cr(III), which ultimately forms stable Cr-Fe hydroxide precipitates via coprecipitation [10,11].

Biological synthesis of nano-FeS, most commonly using SRB to reduce sulfate to S^2−^ which then reacts with Fe^2+^ to form FeS, offers distinct advantages over chemical synthesis. It leverages a wide range of raw material sources (including Fe and sulfate from industrial waste), has low energy requirements, and produces no harmful chemical residues, aligning perfectly with green remediation principles [12,13]. For example, adjusting glucose concentration to 1000 mg/L and applied voltage to 0.2 V at the cathode of a microbial fuel cell (MFC) enabled in situ biosynthesis of smaller, more abundant nano-FeS, which delivered a dramatic boost in Cr(VI) removal efficiency [14]. Even so, biologically synthesized nano-FeS faces significant stability challenges that limit its real-world use: its high surface energy drives severe agglomeration, which reduces specific surface area and eliminates active sites [11,15]. While nZVI alone tends to form micron-sized particles in aqueous solutions, it can maintain nanoscale dispersion when loaded on biochar [15], highlighting the critical need for stabilization strategies for nano-FeS as well. Additionally, nano-FeS is highly susceptible to oxidation to Fe(III) oxides in aerobic environments, losing its reducing activity entirely; studies show the activity of unstabilized nano-FeS drops by roughly 40% after just seven days of air exposure [16]. Coexisting ions in AMD, such as SO_4_^2−^ and Cl^−^, as well as natural organic matter, further compromise stability: SO_4_^2−^ in particular competes with Fe(II) for binding sites, directly reducing Cr(VI) removal efficiency [9,17]. In aerobic conditions, the oxidation half-life of nano-FeS is a mere 2 to 3 h, and agglomeration can reduce its specific surface area by more than 50%, severely limiting its remediation efficiency and service life [18,19]. These stability challenges represent the primary barrier to the practical application of bio-based nano-FeS in AMD remediation.

Humic acid (HA), a core component of natural organic matter, has emerged as a highly effective stabilizer for nanomaterials, owing to its abundant carboxyl and phenolic hydroxyl groups and unique colloidal properties. HA interacts with nanomaterial surfaces via electrostatic interactions, hydrogen bonds, and coordination bonds, forming a stable coating that prevents particle agglomeration [20,21]. Its low cost, wide availability, and environmental friendliness have made it valuable across the petroleum, chemical, construction, healthcare, and environmental protection sectors. For example, HA-stabilized silver nanoparticles (HS-AgNPs) exhibit far superior dispersibility and antioxidant properties compared to their unstabilized counterparts [20]. Over seven days in pH 5–9 environments, HA-stabilized nano-FeS showed an oxidation rate just 15% that of unstabilized samples, with Cr(VI) removal efficiency increasing by more than 30% [19,22]. HA forms stable chelates with Fe^2+^ on nZVI surfaces via carboxyl groups, reducing nZVI particle size from 1000 nm to below 200 nm [19]. For nano-FeS specifically, HA’s hydroxyl groups form hydrogen bonds with S^2−^ on FeS surfaces, while its macromolecular structure creates a protective barrier that delays oxidation [23,24]. In simulated AMD conditions (pH = 4–6, 50 mg/L Cr(VI)), HA-stabilized nano-FeS delivered 40% higher remediation efficiency than unstabilized samples, demonstrating its ability to enhance nanomaterial performance in complex acidic environments [22]. Beyond stabilization, HA boosts Cr(VI) removal performance by providing additional adsorption sites via its functional groups, facilitating Cr(VI) enrichment at the material surface. Quinone structures in HA also act as electron shuttles, accelerating electron transfer from FeS to Cr(VI) and speeding up its reduction to Cr(III) [20,21]. Studies show HA-stabilized nano-FeS achieves roughly 20% higher Cr(VI) removal rates in complex water matrices than unstabilized samples [17]. Furthermore, HA improves the biocompatibility of nano-FeS, reducing its toxicity to environmental microorganisms and promoting biological synergy in remediation systems [14,25]. In short, HA stabilization effectively addresses the core stability challenges of nano-FeS in biological remediation applications.

Previous studies indicate that HA can stabilize chemically synthesized FeS. However, these studies mainly focused on nanomaterials from traditional chemical methods and often overlooked HA’s in situ regulatory mechanisms during biosynthesis. Importantly, existing research has not systematically explained how HA modulates the interfacial reaction pathways of biogenic FeS at the molecular level to achieve efficient and durable immobilization of Cr(VI). This study fills this gap by exploring HA’s role in FeS biosynthesis and revealing HA-mediated interfacial buffering and electronic regulation mechanisms. It proposes a more economical, environmentally friendly, and effective strategy for treating Cr(VI) in AMD compared to traditional chemical precipitation and adsorption techniques. The principal innovations of this work include the following: (1) introducing an HA stabilization strategy into an SRB biosynthetic FeS system for the first time; (2) uncovering the molecular mechanisms by which HA regulates FeS/Cr(VI) interfacial reactions; (3) overcoming the common issues of passivation and short service life seen with conventional materials.

Existing biological remediation literature primarily focuses on the use of HA in the chemical synthesis of FeS nanoparticles, with limited studies addressing its role in biosynthesis. This study aimed to fill this gap by exploring several key aspects: (1) optimizing the biosynthetic conditions for nano-FeS using a combination of single-factor experiments and response surface methodology, with the Cr(VI) removal rate serving as the response variable; (2) investigating the Cr(VI) removal efficiency of the HA-FeS product before and after HA addition to determine the optimal HA concentration; (3) examining the reaction mechanism of Cr(VI) removal by HA-FeS, based on characterization results and comparisons before and after interaction with Cr(VI). Most experiments were conducted in simulated acidic Cr(VI)-containing mine-water conditions, and competitive ions typical of AMD (high sulfate and hardness cations) were not systematically evaluated. Consequently, the reported performance should be interpreted as baseline behavior under controlled conditions; future work will quantify anti-interference performance using representative ionic-strength gradients and major-ion additions to better approximate real AMD matrices. This research addresses the challenges of nano-FeS oxidation and aggregation, proposing a novel strategy for treating Cr(VI) in AMD.

## 2. Results and Discussion

### 2.1. Results of Single-Factor Experiment

1. Dosage of yeast extract powder effects on the preparation of nano-FeS

**Figure 1:**
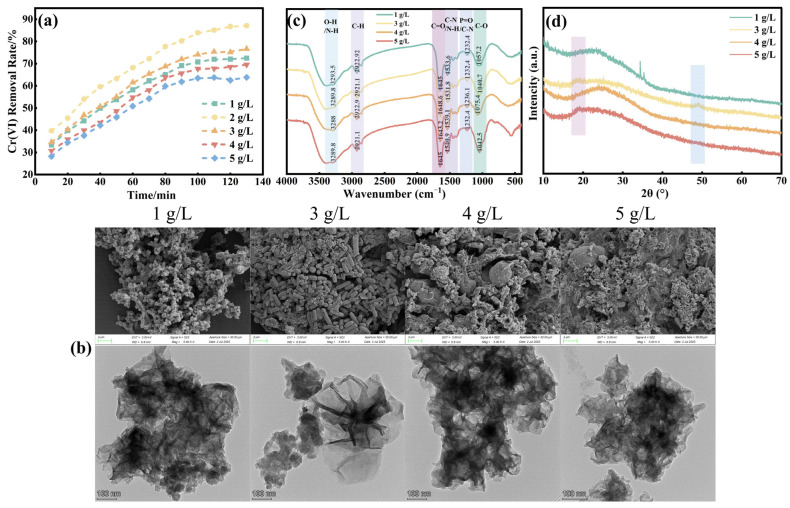
(**a**) Removal of Cr(VI) by bio-nano FeS prepared under different dosage of yeast extract powder; (**b**) The SEM and TEM images illustrate the nano-FeS synthesized using varying amounts of yeast extract powder; (**c**) The FTIR spectra display the nano-FeS produced with different yeast extract powder dosages; (**d**) The XRD patterns reveal the structural characteristics of nano-FeS prepared with differing yeast extract powder quantities.

This research first evaluated the influence of yeast extract powder dosage on FeS biosynthesis and subsequent Cr(VI) removal performance, with dosages ranging from 1 to 5 g/L. As shown in Figure 1a, Cr(VI) removal efficiency exhibited a clear rise-and-fall trend with increasing yeast extract powder dosage, mirroring the trend in FeS production, which peaked at 345 mg/L at 2 g/L before declining to 238 mg/L at 5 g/L.

This trend is directly reflected in the material’s morphology, as shown in the SEM and TEM images in Figure 1b (The detailed magnified microscopic morphology is shown in Supplementary Material Figure S1). At low yeast extract powder dosages, lamellar FeS structures were poorly developed, with severe nanoparticle agglomeration. At 2 g/L, the lamellar structures were most abundant, with markedly improved particle dispersion. As dosage increased beyond 2 g/L, however, lamellar structures diminished, nanoparticle agglomeration intensified, and particles formed more regular short-rod and plate-like shapes. Larger aggregates formed locally, internal pores decreased, and the particle structure became denser—all of which reduce the contact area between the material and Cr(VI), lowering removal efficiency.

FTIR spectra (Figure 1c) revealed clear changes in surface functional groups with varying yeast extract powder dosages. The peak intensity in the 3500–4000 cm^−1^ range (O–H/N–H) fluctuated slightly, with a stronger peak at 1 g/L and a weaker peak at 5 g/L. Peak intensity in the 2900–3000 cm^−1^ range (C–H) initially increased and then decreased with higher yeast dosages, peaking at 3 g/L. Peak deformation broadened, and intensity slightly increased in the 1700–1600 cm^−1^ range (C=O, C–N/C–O), with the 5 g/L curve showing a wider and stronger peak. Peak intensity in the 1000–1300 cm^−1^ range (C–O, P=O) decreased consistently as yeast dosage increased. In the XRD pattern shown in Figure 1d, the overall intensity of the FeS diffraction peaks weakens, with characteristic peaks becoming less sharp and more dispersed, indicating reduced crystallinity of the nano-FeS. In Figure 1d, shaded regions serve only as visual guides to compare broad diffraction features across samples; they are not used for definitive phase identification. Given the low crystallinity of biogenic FeS, XRD is interpreted qualitatively (peak broadening/attenuation trends) rather than as conclusive proof of specific phases.

These observations can be explained by the dual role of yeast extract powder as a carbon source and nutrient for SRB growth, as previously reported by Tang et al. [26]. At 1 g/L, the nutrient supply in the culture medium was insufficient, weakening SRB metabolic activity. This insufficiency likely hindered the synthesis of key enzymes or cofactors in the metabolic pathway, thereby reducing FeS production [26]. Additionally, SRB growth and sulfur production rely on an electron transport chain, and a low yeast extract powder dosage may disrupt electron transport, obstructing sulfide generation. Insufficient nutrients could also alter SRB cell structure, increasing cell membrane permeability, which may destabilize FeS, causing nano-FeS agglomeration and diminishing Cr(VI) removal efficiency.

Previous studies have also shown that yeast extract significantly enhances the bioavailability of metal elements like iron in anaerobic systems: samples with added yeast extract showed higher concentrations of dissolved metals, particularly iron, compared to those without [27]. This suggests that yeast extract powder improves iron solubility and bioavailability, facilitating SRB utilization and FeS formation. However, when the yeast extract powder dosage increased to 5 g/L, the Cr(VI) removal rate dropped sharply to 63.8%. While the sugar components in yeast extract powder serve as a carbon source to promote SRB cell growth, metabolism, and reproduction, increasing dosage from 2 g/L to 5 g/L decreased nano-FeS production by 107 mg/L and reduced Cr(VI) removal by 23.29%. Higher yeast extract powder dosages may lead to the accumulation of intermediate metabolic products, disrupting normal metabolic pathways. This accumulation can imbalance the uniformity and nucleation conditions of FeS precursors, increasing the proportion of amorphous/low-crystalline particles and reducing crystallinity. Moreover, yeast biomolecules like polysaccharides and proteins have binding effects: as yeast dosage increases, the total biomolecule content rises, promoting nano-FeS particle agglomeration. Yeast molecules further regulate nanoparticle aggregation in a fine order, intensifying agglomeration. Consequently, the grain size of nano-FeS decreases with increased yeast dosage, and the internal structure of agglomerates becomes denser, reducing Cr(VI) removal efficiency.

Overall, the amount of yeast extract powder significantly influenced FeS production by SRB: an optimal dosage improved Fe bioavailability and bacterial activity, thereby increasing FeS production. Consequently, 2 g/L of yeast extract powder was selected as the carbon source for subsequent experiments.

2. Fe/S molar ratio effects on the preparation of nano-FeS

**Figure 2:**
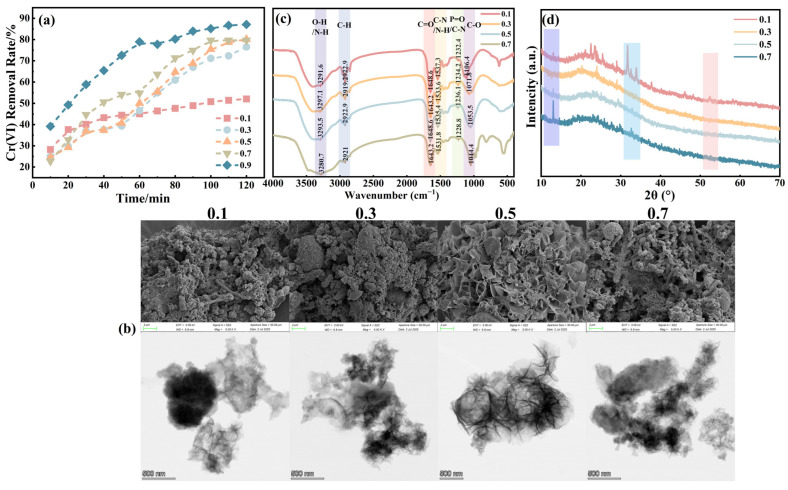
(**a**) Removal of Cr(VI) by bio-nano FeS prepared under different Fe/S molar ratio conditions; (**b**) SEM and TEM images depict nano-FeS synthesized at varying Fe/S molar ratios; (**c**) The FTIR spectra illustrate the nano-FeS produced at these different ratios; (**d**) XRD patterns reveal the structural characteristics of nano-FeS prepared with various Fe/S molar ratios.

Figure 2a illustrates that at Fe/S molar ratios of 0.1, 0.3, 0.5, 0.7, and 0.9, the FeS generation amounts were 183, 274, 310, 329, and 442 mg/L, respectively. Correspondingly, the Cr(VI) removal rates were 52.06%, 76.52%, 80.09%, 79.64%, and 87.23%. As the Fe/S molar ratio increased, the Cr(VI) removal rate also rose. Figure 2b illustrates the transformation of the sample from a dense, amorphous agglomerated block to a “petal-like” or layered nanosheet structure (The detailed magnified microscopic morphology is shown in Supplementary Material Figure S2). This change significantly increases the material’s external specific surface area and the number of edge sites. High-resolution TEM images reveal that particles prepared with a high Fe/S molar ratio have more extended, thinner, and transparent edges. This ultra-thin nanosheet structure reduces the electron transfer path from the bulk phase to the surface. In Figure 2c, FTIR spectra reveal that varying Fe/S ratios systematically altered surface functionalities of biogenic FeS. Increasing Fe/S enhanced O–H/N–H bands (~3290 cm^−1^) while attenuating C–O (1040–1070 cm^−1^), indicating shifts in organic association and Fe–S nucleation pathways. Figure 2d indicates that the overall intensity of FeS diffraction peaks diminished, with decreased sharpness and clarity, a tendency towards diffuse peak shapes, and slight shifts in peak positions.

These trends can be explained by the effects of Fe/S ratio on SRB metabolism and FeS nucleation. When the carbon source in the medium remains constant, a lower Fe/S molar ratio causes substrate competition among SRB. During metabolism, H_2_S concentration rises quickly, reducing SRB’s metabolic and proliferation rates [28]. This decline limits reaction sites for FeS formation. Excess sulfur from SRB metabolism also adsorbs on the FeS surface, promoting crystal growth, reducing nanoscale characteristics, and decreasing Cr(VI) removal efficiency.

Biological nanoscale FeS primarily consists of mackinawite, which has abundant structural defects. Moderate sulfur excess results in smaller particles and a larger specific surface area, enhancing Cr(VI) interaction and surface reaction. However, excessive sulfur forms a sulfur-rich secondary phase (S^0^/polysulfides) on the surface, reducing active Fe(II) sites for electron transfer. The surface may also become weakly alkaline, hindering the adsorption of strongly oxidizing anions like HCrO_4_^−^. Excess sulfur also inhibits thioredoxin reductase activity, affecting FeS synthesis rate and yield. Stoichiometrically, the theoretical Fe:S ratio of FeS is 1:1; when the Fe/S molar ratio approaches one, it increases iron content and promotes FeS synthesis [29]. This balance facilitates reaction, passivation, and regeneration, maintaining continuous reducing capacity. Therefore, an Fe/S molar ratio of 0.9 was selected as the baseline level for further optimization.

3. NH_4_Cl dosage effects on the preparation of nano-FeS

**Figure 3:**
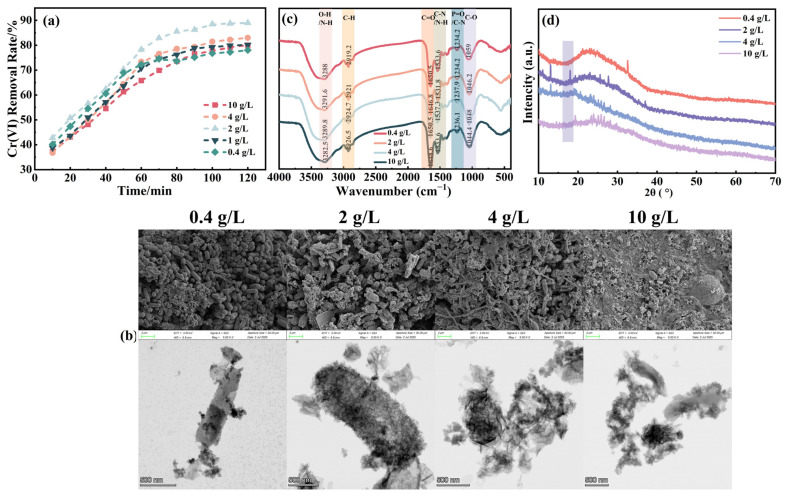
(**a**) Removal of Cr(VI) by bio-nano FeS prepared under different NH_4_Cl dosage; (**b**) SEM and TEM images of nano-FeS prepared with different NH_4_Cl dosage; (**c**) FTIR spectra of nano-FeS prepared with different NH_4_Cl dosage; (**d**) XRD patterns of nano-FeS prepared with different NH_4_Cl dosage.

Figure 3a illustrates the impact of varying NH_4_Cl dosage on the treatment of Cr(VI)-containing acidic wastewater using biological nano FeS. The FeS yields in each system were 318, 412, 456, 378, and 305 mg/L, respectively, with the highest yield of 456 mg/L occurring at an NH_4_Cl dosage of 2 g/L. At 120 min into the reaction, the Cr(VI) removal rate peaked at 89%. SEM and TEM images (Figure 3b) show that increasing the NH_4_Cl dosage from 0.4 g/L to 10 g/L led to a clear shift in particle morphology and dispersion. At 2 g/L, the particle size distribution was narrow, with clear grain boundaries and an ordered structure. As dosage increased beyond 2 g/L, however, nanoparticle agglomeration intensified, with grain boundaries becoming blurred and surfaces becoming disordered (The detailed magnified microscopic morphology is shown in Supplementary Material Figure S3). FTIR spectra (Figure 3c) mirrored these trends: as NH_4_Cl dosage increased, the intensity at 3288 cm^−1^ in the O–H/N–H region first intensified and then decreased, as did the intensity at 2919.2 cm^−1^ in the C–H region. In the C=O/C–N region at 1650.5 cm^−1^, the intensity gradually intensified before weakening, while in the C–O region at 1059 cm^−1^, it consistently weakened. XRD patterns (Figure 3d) showed that with varying NH_4_Cl dosages, the intensity of the FeS diffraction peak diminished, indicating a decrease in nano FeS crystallinity, with the peak shape becoming more dispersed and less sharp.

These observations stem from the dual role of NH_4_Cl as a nitrogen source for SRB metabolism and a modifier of system ionic strength, which significantly influences Cr(VI) removal through nutritional/physiological and salinity/ionic strength effects. When NH_4_Cl concentration is low (<2 g/L), SRB face “nitrogen limitation,” necessitating additional energy expenditure for environmental adaptation. SRB undergo various growth phases—adjustment, logarithmic, stable, and decline [30]—which can divert energy from FeS synthesis. This results in slow nucleation and slow growth of FeS, potentially leading to incomplete sulfidation and inclusion of by-phases like iron (hydrogen) oxides and carbonates. Consequently, effective reaction sites and electron supply per unit mass decrease, slowing Cr(VI) reduction.

At a moderate NH_4_Cl concentration of 2 g/L, cell activity and sulfide production rates increase significantly. Rapid HS^−^ generation enhances system supersaturation, promoting swift nucleation of mackinawite-type FeS with smaller particle sizes, higher specific surface areas, and greater defect densities, facilitating faster and more continuous Cr(VI) removal. Increasing NH_4_Cl dosage from 0.4 to 2 g/L also maintains the system’s ionic strength and pH, promotes the dissolution and activity of yeast-secreted biocomponents, and enhances the vibration signals of functional groups like O–H, C–H, and C=O. This adjustment increases the nucleation rate while decreasing the growth rate of the Fe–S reaction, leading to refined grains and a concentrated particle size distribution, and promoting the formation of regular FeS crystal structures.

Conversely, excessive NH_4_Cl (≥4 g/L) induces osmotic pressure/ionic strength stress. In neutral preparation systems, it may inhibit free ammonia (NH_3_), reducing microbial activity and sulfide production rates, resulting in non-nanoscale FeS and decreased yield. The high ion concentration also disrupts electrostatic repulsion between nanoparticles, damaging the spatial structure of biomolecules and enzyme activity, weakening the vibration signals of biomolecules. Excess Cl^−^ interferes with the binding between biomolecules and nanoparticles, reducing FTIR peak intensity, and disrupts the structural guiding ability of biological templates, causing intensified particle agglomeration and disordered morphology, which affects nanoparticle crystallization conditions. This interference may also lead to the formation of impurities such as Fe(OH)_3_ and Fe_2_O_3_, resulting in decreased crystallinity and peak shape dispersion. Reaction products like Fe(III)/Cr(III) can form a deposition layer (hydroxide/mixed deposition), potentially causing passivation, though systems with ample sulfides, numerous structural defects, and good particle dispersion are less likely to be completely obstructed by a dense passivation layer.

To synthesize FeS that is more nanoscale, more defective, more dispersed, and less susceptible to passivation, a NH_4_Cl concentration of 2 g/L was selected for subsequent optimization experiments.

### 2.2. Analysis of RSM

According to the single-factor test results, the RSM test was carried out, and the test results are shown in Table 1.

According to the results in Table 1, through Box–Behnken experimental calculation, the quadratic polynomial regression model of the preparation of nano-FeS treated Cr(VI) under different factors is obtained as follows:

(1) $$\mathrm{Cr}(\mathrm{VI})\mathrm{Removal}\mathrm{Rate}\left(\%\right)=83.18+3.01A+8.40B+0.97C-2.07\mathrm{AB}-0.38\mathrm{AC}-0.30\mathrm{BC}-4.04A^{2}-5.72B^{2}-4.34C^{2}$$

Analysis of variance was performed on the second-order model, as shown in Table 2.

As can be seen in Table 2, the ANOVA results show that the model F-value is 312.37 and the significance test *p*-value is <0.001, which indicates that the equation is well regressed and the model is extremely significant, which can be used as an alternative to the true point of the test for the prediction of the outcome analysis. The R^2^ of the model = 0.9975, indicating that only 0.0025 of the variance could not be explained by the model. The model has a high degree of fit and a low misfit term, so the experimental results for Cr(VI) removal can be analyzed using this regression equation. The calibration coefficient of determination represents the degree of fit of the model, and the model R^2^_Adj_ = 0.9943, which indicates that the model can solve the response variation of 99.43% response values, so the model fits and regresses well with low error. Also, the model has a test precision of 51.309 (>4), a coefficient of variation of 0.77% (<10%) and a misfit term of 2.10 (>0.05), indicating that the model has a high degree of precision and feasibility, and is suitable for use in analyzing and predicting Cr(VI) removal rates [31]. Therefore, the model is suitable for analyzing and predicting the Cr(VI) removal rate. In the model, the differences in primary terms A and B were highly significant, and the difference in C was significant. The F-values of A, B and C were 208.21, 1616.99 and 21.57, respectively, and the magnitude of the F-values showed that the order of single-factor effects was B > A > C, which indicated that the Fe/S molar ratio had the most significant effect on the removal rate of Cr(VI). The differences in the interaction term AB were significant, and the differences in AC and BC were not significant. The differences in the secondary terms A^2^, B^2^ and C^2^ were extremely significant. In summary, RSM can better simulate the removal of total chromium by FeS nanoparticles prepared by the biological method and provide a more suitable and accurate model.

**Figure 4:**
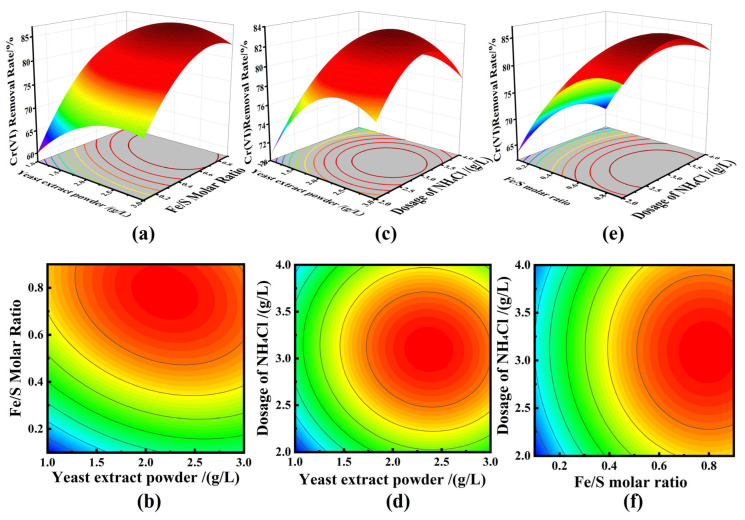
Contour plots and response surface methodology (RSM) diagrams showing the effects of biosynthesis variables on Cr(VI) removal efficiency by biogenic nano-FeS. (**a**) Response surface plot of the interactive effect of yeast extract powder dosage and Fe/S molar ratio on Cr(VI) removal rate; (**b**) Contour plot of the interactive effect of yeast ex-tract powder dosage and Fe/S molar ratio on Cr(VI) removal rate; (**c**) Response surface plot of the interactive effect of yeast extract powder dosage and NH_4_Cl dosage on Cr(VI) removal rate; (**d**) Contour plot of the interactive effect of yeast extract powder dosage and NH4Cl dosage on Cr(VI) removal rate; (**e**) Response surface plot of the interactive effect of Fe/S molar ratio and NH_4_Cl dosage on Cr(VI) removal rate; (**f**) Contour plot of the interactive effect of Fe/S molar ratio and NH_4_Cl dosage on Cr(VI) removal rate.

This research utilized Design-Expert 8.0.6 software for function analysis and optimization. The Box–Behnken Design (BBD) method was employed to evaluate the impact of yeast extract powder dosage, Fe/S molar ratio, and NH_4_Cl dosage on Cr(VI) removal using nano-FeS. BBD efficiently fits a quadratic model with fewer experiments, avoiding extreme factor combinations that could destabilize SRB-mediated biosynthesis. This approach is ideal for response surface method optimization. This study predicted the optimal conditions for the biogenic synthesis of nano-FeS and its removal efficiency. Figure 4 displays the RSM and contour plots.

The RSM plots in Figure 4a show the effect of yeast extract powder dosage and Fe/S molar ratio on the removal of Cr(VI) by biologically prepared FeS nanoparticles when the NH_4_Cl dosage is 3 g/L. The removal of Cr(VI) increased with the increase in Fe/S molar ratio at a constant yeast dosage. When the Fe/S molar ratio was constant, the removal of Cr(VI) increased and then decreased with the increase in yeast dosage. The *p*-value of the interaction between yeast dosage and Fe/S molar ratio was 0.0002 (<0.001), indicating that the interaction was highly significant.

The RSM plots of the effect of yeast extract powder dosage and NH_4_Cl dosage on the removal of Cr(VI) by the prepared nano FeS when the Fe/S molar ratio was 0.5 are shown in Figure 4c. From the figure, it can be seen that the removal rate of Cr(VI) increased with the increase in NH_4_Cl dosage when the yeast extract powder dosage was constant; when the NH_4_Cl dosage was constant, the removal rate of Cr(VI) still showed an increasing trend with the increase in yeast extract powder dosage. This suggests that the nano-FeS prepared by moderately increasing the dosage of carbon and nitrogen sources has a promoting effect on the removal of Cr(VI). The *p*-value for the interaction between dosage of yeast and NH_4_Cl dosage was 0.2337 (>0.05), indicating an interaction, but not significant.

The RSM plots in Figure 4e depict the effects of Fe/S molar ratio and NH_4_Cl dosage on the Cr(VI) removal by the prepared nano FeS when 2 g/L yeast extract powder was added. When the Fe/S molar ratio was constant, the Cr(VI) removal increased with the increase in NH_4_Cl dosage. When the mass ratio of NH_4_Cl dosage was constant, the removal rate of Cr(VI) increased significantly with the increase in Fe/S molar ratio. The *p*-value of the interaction between the Fe/S molar ratio and the NH_4_Cl dosage was 0.3436 (>0.05), thus indicating that the correlation between the two was not significant.

Figure 4b,d,f are the contour plots of the interactive effects of yeast extract powder dosage and Fe/S molar ratio, yeast extract powder dosage and NH_4_Cl dosage, and Fe/S molar ratio and NH_4_Cl dosage on the Cr(VI) removal rate, respectively. The contour lines in Figure 4b are more densely distributed on the side of the Fe/S molar ratio factor, indicating that the Fe/S molar ratio plays a dominant role in the interactive influence of the two factors on the Cr(VI) removal rate. According to the variance results of the response value regression model, the *p*-value of the interaction between yeast extract powder dosage and Fe/S molar ratio is 0.0002, with *p* < 0.001, indicating that there is an extremely significant interactive effect of the two factors on the Cr(VI) removal rate. The contour lines in Figure 4d are more densely distributed on the side of the yeast extract powder dosage factor, indicating that the yeast extract powder dosage plays a dominant role in the interactive influence of the two factors on the Cr(VI) removal rate. According to the variance results of the response value regression model, the *p*-value of the interaction between yeast extract powder dosage and NH_4_Cl dosage is 0.2337, with *p* > 0.05, indicating that there is an interactive effect of the two factors on the Cr(VI) removal rate, but it is not significant. The contour lines in Figure 4f are more densely distributed on the side of the Fe/S molar ratio factor, indicating that the Fe/S molar ratio plays a dominant role in the interactive influence of the two factors on the Cr(VI) removal rate. According to the variance results of the response value regression model, the *p*-value of the interaction between Fe/S molar ratio and NH_4_Cl dosage is 0.3436, with *p* > 0.05, indicating that there is an interactive effect of the two factors on the Cr(VI) removal rate, but it is not significant. Combined with the regression model variance, it can be concluded that the strength of the interaction between the three factors is as follows: yeast dosage and Fe/S molar ratio > yeast extract powder dosage and NH_4_Cl dosage > Fe/S molar ratio and NH_4_Cl dosage.

Table 3 shows the model-predicted optimal parameters: yeast extract powder dosage of 2.19 g/L, Fe/S molar ratio of 0.78, and NH_4_Cl dosage of 3.08 g/L. These were adjusted to practical values (yeast extract powder dosage: 2.2 g/L; Fe/S molar ratio: 0.8; NH_4_Cl dosage: 3.1 g/L), yielding optimal conditions.

### 2.3. Effects of HA Dosage on Nano-HA-FeS Formation and Performance

**Figure 5:**
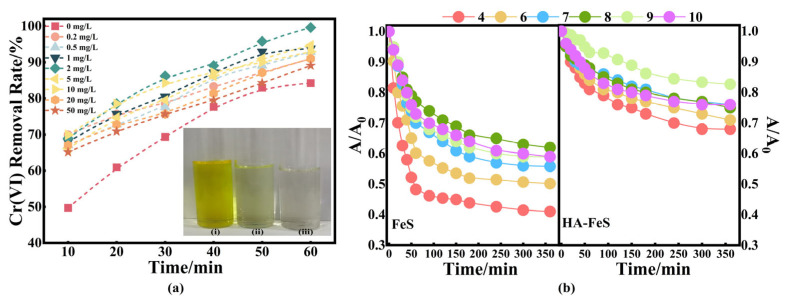
(**a**) Time-dependent Cr(VI) removal by FeS synthesized with different HA dosages (colored curves denote HA concentration in the synthesis medium, mg/L). Insets: (**i**) Untreated Cr(VI) contaminated water sample; (**ii**) FeS treated water sample; (**iii**) HA-FeS treated water sample prepared by adding 2 mg/L HA; (**b**) Dispersion/settling stability expressed as A/A_0_ versus time for FeS and HA-FeS.

After determining the optimal conditions for the biosynthesis of FeS, the effects of the addition amount of HA on the FeS yield and Cr(VI) removal performance were investigated. The results are shown in Figure 5a. Without HA addition, the FeS yield was 710.75 mg/L, with 84.25% Cr(VI) removal after 60 min. Moderate HA addition markedly enhanced both FeS yield and reactivity, with yield reaching a maximum of 1096.26 mg/L at 2 mg/L HA, and nearly complete Cr(VI) removal (99.62%) was achieved within 60 min at this same dosage. This dramatic performance improvement is consistent with the improved stability of HA-FeS observed in pre-reaction stability tests, where HA-FeS maintained substantially higher A/A_0_ values over time compared to bare FeS, indicating suppressed aggregation and oxidative degradation (Figure 5b). Similar non-linear effects of natural organic matter on biogenic mineral formation have been reported previously [32,33].

Mechanistically, HA plays a dual role in regulating Cr(VI) removal by biogenic FeS. First, HA adsorbs onto FeS surfaces through coordination with oxygen-containing functional groups (–COOH and –OH), forming an organic coating that enhances colloidal stability and preserves reactive Fe^2+^ and S^2−^ sites under acidic conditions [34]. Second, humic substances can function as electron shuttles, facilitating electron transfer from FeS to Cr(VI) and accelerating its reduction to Cr(III) [35,36]. These effects collectively explain the rapid decolorization observed in the HA-FeS-treated water (Figure 5a(iii)), compared to the partially clarified solution treated with unstabilized FeS (Figure 5a(ii)) and the bright yellow Cr(VI)-rich raw wastewater (Figure 5a(i)).

However, when the HA concentration exceeded the optimal range (≥10 mg/L), Cr(VI) removal efficiency decreased slightly to 89.01–92.91%, despite continued FeS production. This decline can be attributed to two key factors: first, excessive HA coverage on FeS surfaces may block electron-donating sites and hinder direct contact between Cr(VI) and the FeS core; second, dissolved HA can strongly complex Cr(III), suppressing its precipitation as stable sulfide or mixed Fe–Cr mineral phases [37]. FeS yield also gradually declined at HA concentrations ≥ 5 mg/L, suggesting that excessive HA may inhibit microbial FeS mineralization by complexing Fe^2+^ or altering microbial metabolic pathways.

An optimal HA concentration of 2 mg/L effectively balances FeS yield, stability, and interfacial reactivity, resulting in efficient and sustained Cr(VI) removal. These results demonstrate that controlled HA stabilization not only enhances the production and stability of biogenic FeS but also fundamentally alters the Cr(VI) reduction pathway, enabling rapid and near-complete detoxification of acidic Cr(VI)-contaminated wastewater.

### 2.4. Cr(VI) Removal Kinetics

**Figure 6:**
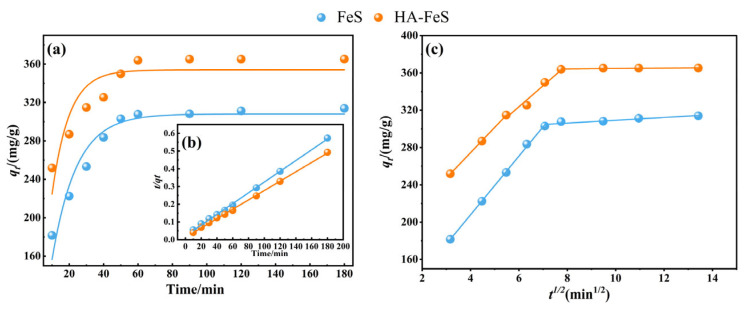
Curve fitting of kinetic models was conducted to analyze the removal of Cr(VI) using biological nano-FeS and HA-FeS. (**a**) The models included the pseudo-first-order kinetic model, (**b**) the pseudo-second-order kinetic model, and (**c**) the intraparticle diffusion model.

Figure 6 shows the removal kinetics of Cr(VI) by pristine FeS and HA-FeS. Both materials exhibited non-linear kinetics characterized by a rapid initial rise in Cr(VI) removal, followed by a gradual approach to a plateau. Initially, abundant active sites and low interfacial mass transfer resistance facilitate rapid reaction; over time, however, occupation of surface sites, formation of product films or deposits, and increased diffusion resistance within pores slow the reaction rate. This behavior aligns with the typical adsorption–reduction-precipitation/coprecipitation process observed in Cr(VI) treatment using zero-valent iron/FeS systems [38].

Three kinetic models were used to fit the experimental data, with fitting parameters shown in Table 4. Pseudo-first-order kinetics fit pristine FeS moderately well (R^2^ = 0.9231), but poorly fit HA-FeS (R^2^ = 0.7782), indicating that the rate change in HA-stabilized material cannot be explained solely by concentration-driven first-order kinetics. In contrast, the pseudo-second-order kinetic model yielded a correlation coefficient near one for both materials (FeS: R^2^ = 0.9985; HA-FeS: R^2^ = 0.9991), with the calculated equilibrium capacity of HA-FeS increasing by approximately 15% compared to pristine FeS. These findings suggest that HA-FeS not only boosts the final removal amount but also enhances the overall apparent reaction rate. Given HA’s effects on nanoparticle dispersion, stabilization, and agglomeration inhibition, it likely improves process kinetics by expanding the accessible active interface and delaying particle deactivation. The excellent fit of pseudo-second-order kinetics often implies that interface reactions, such as surface complexation and electron transfer, are involved in rate control [39]. However, this model is fundamentally empirical and should not be directly equated with pure chemical reaction rate control based solely on R^2^ values. Therefore, this study also employed the intraparticle diffusion model to distinguish the mass transfer steps involved in the reaction process.

The intraparticle diffusion model fitting results (Figure 6c) show a notable reduction in the diffusion rate constant in the later reaction stage for both materials (FeS: *K_p_*_3_ = 1.5145; HA-FeS: *K_p_*_3_ = 0.2109), alongside a significant drop in R^2^ (FeS: 0.8530; HA-FeS: 0.3878). This indicates that the system is increasingly constrained by intra-pore diffusion, depletion of active sites, and heightened mass transfer resistance due to reaction product deposition in the later stages [40]. Crucially, the intraparticle diffusion model plot is segmented and does not intersect the origin, indicating that intraparticle diffusion is not the sole rate-controlling step; external film diffusion and interface reactions also play significant roles [41].

Based on the evidence from the three model types, a more realistic mechanistic understanding of the system emerges: Cr(VI) preferentially adsorbs onto the surface of FeS or HA-FeS in the aqueous phase, where rapid electron-transfer reduction occurs. The resulting Cr(III) is then immobilized through precipitation/coprecipitation or surface complexation [42]. Over time, enhanced diffusion and deposition effects reduce the reaction rate. Through quantitative comparison of R^2^ and rate constants, this study supports using the pseudo-second-order kinetic model to describe the system’s macroscopic kinetic behavior. The segmented characteristics of the intraparticle diffusion model suggest that membrane diffusion and rapid surface reactions dominate the initial stage, while intraparticle diffusion and mass transfer resistance from product film/deposition become limiting factors in later stages [43]. Thus, the study provides a traceable kinetic evidence chain at the mechanistic level for the improved Cr(VI) removal performance achieved through HA stabilization.

### 2.5. Adsorption Isotherm Behavior

**Figure 7:**
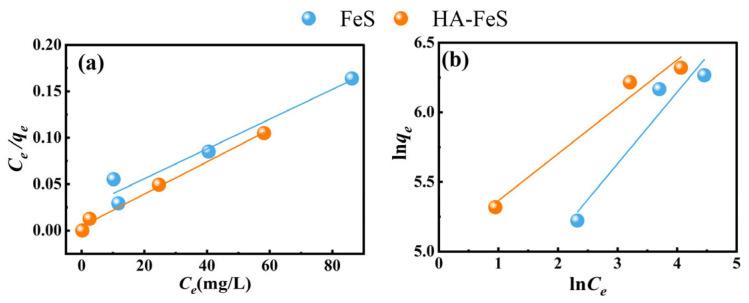
Adsorption isotherm models for Cr(VI) on biological nano-FeS and HA-FeS were evaluated. The Langmuir model (**a**) and the Freundlich model (**b**) were fitted to the data.

The adsorption isotherm behavior of Cr(VI) on pristine FeS and HA-FeS was evaluated using the Langmuir and the Freundlich models. Figure 7 presents the linear fitting results, while Table 5 summarizes the fitting parameters. The Langmuir model, which assumes adsorption occurs on a homogeneous surface with uniform energy active sites (each accommodating one adsorbate molecule), showed a strong linear correlation for both materials, indicating it effectively describes the adsorption behavior within the tested concentration range. This strong fit suggests that Cr(VI) adsorption on these materials is primarily influenced by limited, saturable surface sites rather than multilayer stacking.

Notably, the slope and intercept of HA-FeS’s fitting line differ significantly from those of pristine FeS, indicating variations in theoretical maximum adsorption capacity and affinity constant. HA-FeS exhibits a stronger adsorption response at low-to-medium equilibrium concentrations, implying a more significant increase in adsorption with unit concentration changes, which typically reflects higher surface affinity. The Freundlich model, which highlights the non-uniformity of surface energy distribution and the potential for multilayer adsorption, also fits the experimental data but exhibits weaker linearity than the Langmuir model, particularly at high concentrations where deviations are more pronounced. Although the ln *q_e_* versus ln *C_e_* relationship for both materials appears approximately linear, the data points show more scatter compared to the Langmuir fit, suggesting that surface non-uniformity is not the primary factor governing adsorption. However, the n values in the Freundlich parameters exceed one for both materials, indicating favorable adsorption and a strong spontaneous adsorption tendency of Cr(VI) on FeS and HA-FeS surfaces.

Overall, the Langmuir model better describes the adsorption behavior of both materials compared to the Freundlich model. The HA-FeS system achieves an R^2^ of 0.9904 for the Langmuir model, significantly surpassing the Freundlich model’s R^2^ of 0.9400; similarly, pristine FeS shows a Langmuir R^2^ of 0.9385, outperforming the Freundlich model’s R^2^ of 0.8581. These findings suggest that within the studied concentration range, Cr(VI) adsorption on both materials is a favorable chemical process, more closely resembling monolayer coverage than a multilayer effect. Yao [44] reported that the maximum Cr(VI) removal capacity of HA-FeS reaches 675.03 mg/g, 2.35 times higher than unmodified FeS, aligning with our results. This enhancement is due to HA’s dispersion and stabilization effect on nano-FeS: the hydrated dynamic diameter of HA-FeS particles, reported as 235–241 nm, is much smaller than that of free FeS [44]. HA’s carboxyl and phenolic hydroxyl groups also provide additional coordination sites for Cr(VI) [45]. The high k_L value further indicates a stronger binding tendency of HA-FeS for Cr(VI). Thus, HA increases effective adsorption capacity by preventing FeS agglomeration and boosting the effective specific surface area, while also introducing new adsorption sites that enhance the adsorption rate and affinity for Cr(VI).

It is worth noting that the theoretical maximum adsorption capacity of pristine FeS (621.1180 mg/g) is slightly higher than that of HA-FeS (574.7126 mg/g). This finding indicates that while HA modification improves dispersion and interfacial stability, it may cover some active sites of FeS or engage in complexation, thus limiting the theoretical monolayer capacity at high concentrations. Similar effects have been observed in organic-coated nanomaterials, where stabilizers enhance colloidal stability but may shield surface-active centers [44]. However, the capacity difference between FeS and HA-FeS is not substantial, suggesting that HA modification does not significantly diminish FeS’s intrinsic reduction or adsorption capabilities, and the enhanced affinity and reactivity of HA-FeS make it more effective for practical AMD treatment applications.

### 2.6. Characterization Analysis

1. Morphology and zeta potential analysis

**Figure 8:**
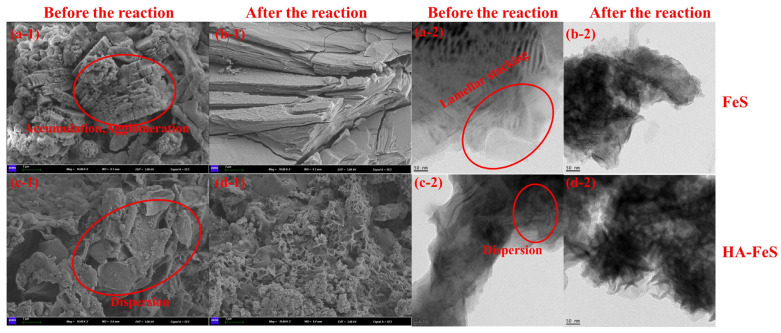
SEM and TEM images of nano FeS and HA-FeS before and after reaction. (**a-1**) SEM image of biological nano-FeS before the reaction; (**b-1**) SEM image of biological nano-FeS after the reaction; (**c-1**) SEM image of HA-FeS before the reaction; (**d-1**) SEM image of HA-FeS after the reaction; (**a-2**) TEM image of biological nano-FeS before the reaction; (**b-2**) TEM image of biological nano-FeS after the reaction; (**c-2**) TEM image of HA-FeS before the reaction; (**d-2**) TEM image of HA-FeS after the reaction.

SEM and TEM (The detailed magnified microscopic morphology is shown in Supplementary Material Figure S4) were used to observe the micro-morphological changes in pristine FeS and HA-FeS before and after reacting with Cr(VI), with zeta potential values measured at pH 4, 6, 7, 8, and 10 shown in Table 6. Prior to the reaction, pristine FeS appeared distinctly agglomerated, with tightly stacked lamellar particles visible in SEM and TEM (Figure 8(a-1,a-2)). This stacking mode, while facilitating local electron transfer, significantly reduces the effective specific surface area of the material. The zeta potential data further elucidate this phenomenon: at pH 4 (the pH of our simulated AMD), pristine FeS exhibited a low surface charge of −8.32 mV, which is insufficient to prevent particle aggregation via electrostatic repulsion [46]. As the pH increased to 10, the potential became more negative, decreasing to −28.67 mV, which enhances dispersion.

In stark contrast, HA-FeS displayed a loose flocculent structure before the reaction (Figure 8(c-1)), with disordered and highly dispersed lamellae in the TEM image (Figure 8(c-2)). This morphology directly correlates with changes in surface electrical properties: at pH 4, HA-FeS had a zeta potential of +3.22 mV, becoming more negative between pH 6 and 10. This suggests that the carboxyl and phenolic hydroxyl groups introduced by HA undergo protonation above neutral pH, enhancing electrostatic repulsion and spatial resistance between particles [47]. Consequently, HA-FeS maintains better dispersion across a broader pH range, offering a more accessible reaction interface for Cr(VI).

After reacting with Cr(VI), the morphology of pristine FeS changed dramatically. Figure 8(b-1) illustrates that the original agglomerates strip along the laminar direction, forming a lamellar fracture structure. In the TEM image (Figure 8(b-2)), the laminar stripes vanish completely, replaced by a dense dark area, indicating the destruction of the FeS lattice and coverage by reaction products due to Cr(VI)’s strong oxidation. Previous studies have shown that FeS readily undergoes oxidation from Fe(II)/S(-II) to Fe(III) and S^0^ in acidic Cr(VI) environments, often leading to structural collapse due to rapid electron transfer [48]. In contrast, the HA-FeS reaction preserves a continuous porous skeleton, as shown in Figure 8(d-1). The lamellar profile remains visible in the TEM image (Figure 8(d-2)), displaying only surface roughening and product deposition, with no large-scale structural disintegration. The higher absolute zeta potential value of HA-FeS also means particles are less prone to re-agglomeration during the reaction, so precipitates distribute more uniformly at the interface, preventing the formation of a dense passivation layer that would block further reaction.

2. FTIR and XRD analysis

**Figure 9:**
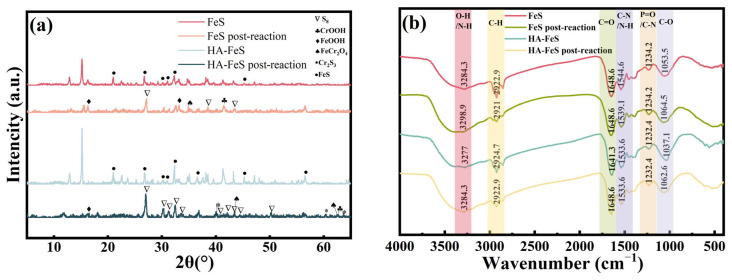
(**a**) FTIR images of nano-FeS and HA-FeS before and after reaction; (**b**) XRD images of nano FeS and HA-FeS before and after reaction.

**Figure 10:**
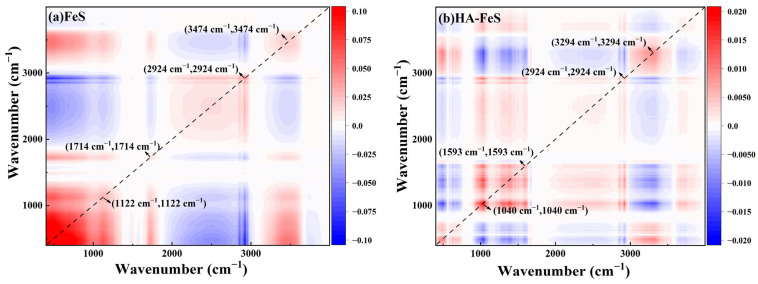
(**a**) 2D-COS characteristics of biologically synthesized FeS before and after the reaction with Cr(VI); (**b**) 2D-COS characteristics of HA-FeS before and after the reaction with Cr(VI).

FTIR spectroscopy was used to identify changes in surface functional groups before and after reaction with Cr(VI), with results shown in Figure 9a. The FTIR spectrum of pristine FeS revealed a broad peak at 3284.3 cm^−1^, indicating –OH/NH stretching vibrations. Peaks between 2921 and 2929 cm^−1^ were attributed to C–H vibrations, while peaks at 1648.6 cm^−1^ and 1539.1 cm^−1^ corresponded to C=O bending vibrations (or adsorbed water) and C–N/–NH vibrations, respectively. The peak at 1053.5 cm^−1^ was associated with C–O vibrations. After reacting with Cr(VI), the peak at 1648.6 cm^−1^ intensified and broadened, while the peak at 1053.5 cm^−1^ shifted slightly to approximately 1064.5 cm^−1^. This indicated an increase in surface hydroxyl and oxygen-containing functional groups, aligning with the oxidation of Fe(II) to Fe(III) hydroxides, which typically serve as carriers for Cr(III) adsorption or co-precipitation [49]. In the 2D-COS synchronous plot (Figure 10), the autocorrelation peaks at 1714 cm^−1^ and 1122 cm^−1^ were prominent, indicating a significant response of carbonyl and C–O structures to the reaction. The sequence of asynchronous signals suggested that changes in oxygen-containing functional groups preceded those in the C–H region, supporting the “surface adsorption–reduction–complexation immobilization” reaction sequence [50].

The spectral characteristics of HA-FeS exhibited a distinctly different evolution trend. Before the reaction, its FTIR spectrum showed distinct peaks at 3277.0 cm^−1^ (–OH/–NH), 2924.7 cm^−1^ (C–H), 1641.3 cm^−1^ (C=O), 1533.6 cm^−1^ (aromatic C=C or C–N), and 1062.6 cm^−1^ (C–O), confirming the successful introduction of HA onto the FeS surface. Post-reaction, the C=O peak slightly red-shifted from 1641.3 cm^−1^ to 1648.6 cm^−1^, and the peak at 1062.6 cm^−1^ shifted to 1037.1 cm^−1^ with decreased intensity, providing direct evidence that carboxyl and phenolic hydroxyl groups from HA participated in metal coordination during the reaction [51]. In the 2D-COS plot (Figure 10), significant autocorrelation peaks at 1593 cm^−1^ and 1040 cm^−1^, positively correlated with 3294 cm^−1^, indicated a synergistic change between oxygen-containing functional groups and the hydroxyl environment. This synergistic response typically reflects structural rearrangements of organic ligands during metal ion binding, further confirming that HA-derived oxygen-containing functional groups play a central role in the interfacial interactions during Cr(VI) removal.

XRD was used to assess the mineral phase transformation of the materials before and after reaction with Cr(VI), with results shown in Figure 9b. After reaction with Cr(VI), the characteristic diffraction peaks of pristine FeS were significantly weakened, indicating extensive oxidative transformation of the FeS structure. Meanwhile, diffraction signals for S_8_ and FeOOH appeared in the 2θ ≈ 26–28° and 31–33° regions, indicating a dissolution-reprecipitation transformation of FeS under acidic conditions [48]. Notably, weak peaks for CrOOH emerged near 2θ ≈36° and 41°, confirming that Cr(VI) was reduced to Cr(III) and deposited as hydroxide [52]. Individual peak positions were close to the standard card of FeCr_2_O_4_ (spinel); however, without XPS or Mössbauer evidence, the formation of a stable spinel structure cannot be confirmed, and is therefore described as a possible local Fe–Cr mixed-oxide structure.

After the reaction of HA-FeS with Cr(VI), the main FeS peak attenuated far less than that of bare FeS, indicating that the HA coating slowed the excessive oxidation of FeS and preserved the material’s reactive core. Simultaneously, characteristic peaks of FeOOH, CrOOH, and a small amount of Cr_2_S_3_ (2θ ≈ 30–32°, 43–45°) were detected. The presence of Cr_2_S_3_ suggests that in the sulfide-rich environment provided by FeS, part of Cr(III) may combine with S^2−^ to form a chromium sulfide phase, which has low solubility under reducing conditions and further enhances the immobilization of Cr. Additionally, new peaks appeared near 2θ ≈ 40–45° and 60°, with some positions close to the diffraction angles of Cr_2_S_3_ or Fe–Cr composite oxides. Compared to the pristine FeS system, the S_8_ peak (around 28–29°) was less prominent in the HA-FeS system, suggesting that the oxidation path of S^2−^ was more restricted, and elemental sulfur might be stabilized in the organic matrix [53]. In the HA-FeS system, the CrOOH peak was more diffuse and had lower crystallinity compared to the FeS system, indicating that Cr(III) is more likely to be fixed on the surface in an amorphous or organically complexed state. The multidentate coordination sites provided by HA molecules enhance the complexation stability of Cr(III), thereby reducing the risk of its remigration in an acidic environment.

A comprehensive analysis of FTIR and XRD results reliably confirms that Cr(VI) is reduced to Cr(III) during the reaction. The primary destinations of Cr(III) include the following: (i) co-precipitation with Fe(III) to form Cr(III)-Fe(III) hydroxyoxides [54]; (ii) partial formation of low-solubility Cr(OH)_3_ or CrOOH [55]; (iii) potential generation of sulfides like Cr_2_S_3_ in sulfur-rich environments [52]. These phases exhibit low solubility and high stability under neutral to weakly acidic conditions, though they may re-release Cr under strongly acidic or oxidative conditions. While the reduction and precipitation of Cr(VI) in FeS systems are well-documented, debates persist regarding how different interface structures influence reaction pathways and product stability. This study uses FTIR, XRD, and 2D-COS to compare the reaction processes of biologically synthesized nano-FeS and HA-stabilized HA-FeS in simulated AMD, examining chromium’s final state and structural stability. Due to the absence of XPS testing, discussions on the Fe–Cr spinel structure are based on diffraction evidence and remain speculative without definitive confirmation.

### 2.7. Mechanistic Insight into Cr(VI) Removal by FeS and HA-FeS

In AMD, Cr(VI)’s high mobility and strong oxidizing properties mean that effective remediation requires both reduction to less toxic Cr(III) and stable solid-phase immobilization to prevent re-mobilization. For pristine bio-nano FeS, a typical Fe(II)-containing sulfide, Cr(VI) removal occurs primarily via electron transfer: surface Fe(II) or S^2−^ donates electrons to Cr(VI), converting it to the more stable and less toxic Cr(III). In this study, the weakening of FeS diffraction peaks and the emergence of new phases like FeOOH, S_8_, and CrOOH in the pristine FeS system confirm that Fe(II) oxidizes to Fe(III), while Cr(VI) reduces and deposits as a hydroxide. Cr(III) typically forms low-solubility Cr(OH)_3_ or CrOOH at pH 4–7, offering greater environmental stability than Cr(VI). However, in the pristine FeS system, Cr(VI) immobilization relies primarily on inorganic precipitation and co-precipitation: Cr(III) co-precipitates with Fe(III) hydroxide or forms an amorphous Cr(III) hydroxide layer on the material surface. While this immobilizes Cr(VI), the dense hydroxide layer also impedes further electron transfer, leading to surface passivation, rapid deactivation of the material, and a risk of Cr re-dissolution under acidic conditions.

After HA stabilization, the reaction pathway exhibits complex interfacial regulation, fundamentally altering the Cr(VI) removal and immobilization process. The HA molecule, rich in carboxyl and phenolic hydroxyl groups, coordinates with Fe sites on the FeS surface, creating a multidentate complexing environment for Cr(III). The redshift and intensity changes in the C=O and C–O peaks in the FTIR confirm the involvement of these functional groups in metal binding. XRD results showing signals of CrOOH and traces of Cr_2_S_3_ suggest that Cr(III) exists in the HA-FeS system in three main forms: co-precipitation with Fe(III) [56], formation of low-solubility sulfide with sulfur [53], and immobilization by organic ligand complexes [57]. Chromium sulfide’s low solubility in reducing environments, combined with the strong organic complexation provided by HA, minimizes the risk of Cr re-release under acidic conditions. This “mineral–organic complex immobilization” mode also explains the relatively loose structure of HA-FeS after reaction, and the partial persistence of the main FeS peak, indicating that HA buffers the rapid oxidation of FeS and preserves the material’s reactive sites for sustained reaction.

The XRD peaks for Fe–Cr spinels, such as FeCr_2_O_4_, closely match standard reference cards. However, without conducting XPS or valence resolution analyses, it is advisable to suggest only the possible existence of a localized Fe–Cr mixed-oxide structure rather than confirming the formation of a stable spinel phase.

**Figure 11:**
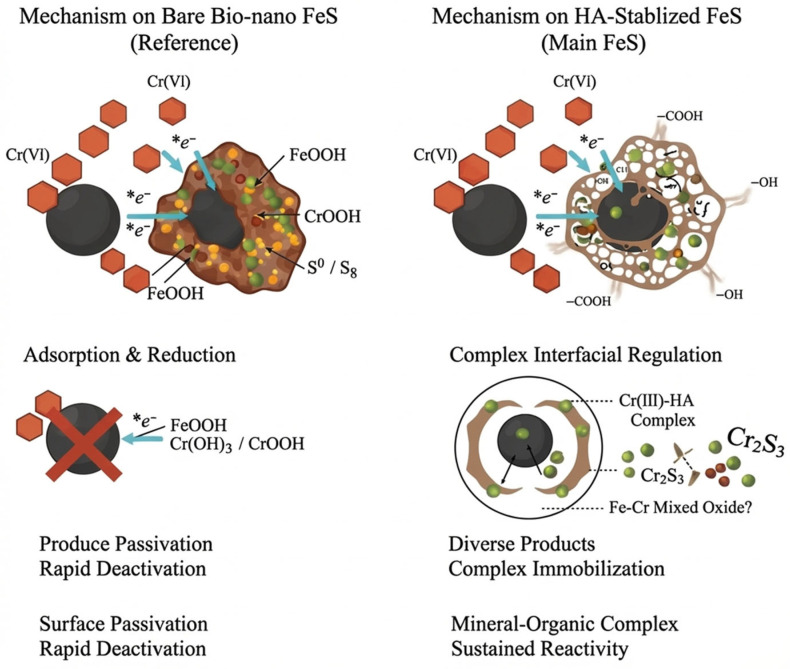
Mechanistic Insight into Cr(VI) Removal by FeS and HA-FeS.

Overall, the core difference between the two systems lies in their immobilization mechanisms and long-term reactivity: the pristine FeS system is prone to deactivation due to surface passivation, while the HA-FeS system retains reactivity by delaying structural transformations through organic encapsulation [58]. The HA-FeS system incorporates organic complexation and interfacial stabilization, creating a more complex and potentially more stable solid-phase environment for Cr(III). Long-term stability of the Cr-bearing solid products under acidic leaching conditions was not evaluated in this dataset. Therefore, statements regarding ‘durable’ immobilization are framed conservatively, and future work will include standardized leaching tests of post-reaction precipitates (acidic pH conditions representative of AMD) with monitoring of dissolved total Cr and Cr(VI) to assess potential re-dissolution and/or re-oxidation. The proposed mechanistic pathways for Cr(VI) removal by pristine FeS and HA-stabilized FeS are summarized in Figure 11.

## 3. Materials and Methods

### 3.1. Materials

K_2_HPO_4_, (NH_4_)_2_Fe(SO_4_)_2_·6H_2_O, MgSO_4_·7H_2_O, and NH_4_Cl were obtained from Sinopharm Chemical Reagent Co., Ltd. (Beijing, China); Na_2_SO_4_ and CaCl_2_·H_2_O were purchased from Macklin Biochemical Technology Co., Ltd. (Beijing, China). K_2_Cr_2_O_7_ was purchased from Quanrui Reagent Co. Ltd. (Jinzhou, China). HA was extracted from lignite collected from a mine in the Baiyinhua mining area (West Ujimqin Banner, Xilingol League, Inner Mongolia Autonomous Region, China). To obtain a Cr(VI) stock solution at a concentration of 1000 mg/L, 2.829 g of dried K_2_Cr_2_O_7_ was dissolved in 1 L of distilled water. All chemicals used in this study were analytical grade or higher. All solutions were prepared using deionized water produced by a Millipore Milli-Q system (Shenyang, China).

### 3.2. Experimental Procedures

#### 3.2.1. Biogenic Nano-FeS Preparation

The SRB used in the experiments were from laboratory-preserved strains, which were connected to the modified Starkey-type medium and cultured in a (30 ± 1) °C constant temperature incubator for enrichment, and the medium was changed every 7 days.

#### 3.2.2. Static Experiment

The single-factor test method was used to examine the effects of reaction Yeast dosage (1, 2, 3, 4 and 5 g/L), Fe/S molar ratio (0.1, 0.3, 0.5, 0.7 and 0.9) and NH_4_Cl dosage (0.4, 1, 2, 4 and 10 g/L) on the preparation of nano-FeS with Cr(VI) in chromium-containing wastewater as the evaluation index. The experiment was set up with 2 groups of parallel experiments. 1 g of nano-FeS centrifugal sediment was weighed and added to 100 mL of acidic chromium-containing wastewater at a pH of 4 with a preliminary Cr(VI) mass concentration of 100 mg/L. The samples were placed in a thermostatic shaking chamber at 30 °C for 1 h at a speed of 150 r/min. Within 1 h of the reaction, samples were taken every 10 min gap. The concentration of Cr(VI) was determined after passing through a 0.22 μm microporous membrane. Cr(VI) concentration was calculated by diphenylcarbazide spectrophotometry (GB 7467-1987) [59].

#### 3.2.3. Optimization of Nano-FeS Preparation Method

On the basis of one-way experiments, the RSM method was used to determine the optimal preparation conditions for the synthesis of FeS nanoparticles by bioprecipitation, and the Box–Behnken model was used to design the RSM optimization experiments. The mass concentration of Cr(VI) in water samples was determined when the reaction time was 60 min. The data were analyzed using Design-Expert 8.0.6 software and the experimental factor codes and levels are shown in Table 7.

The Design-Expert 8.0.6 software is used for fitting, and the experimental model is used to represent the changing relationship between the response value and the factor, as shown in the following formula:

(2) $$Y=\beta_{0}+\sum_{i=1}^{k}\beta_{i}X_{i}+\sum_{i=1}^{k}\beta_{ii}X_{i}^{2}+\sum_{i<j}\beta_{ij}X_{i}X_{j}+\varepsilon$$

*Y*—the system response value;
*β*_0_—the offset term coefficient;
*β_i_*—the linear offset coefficient;
*β_ii_*—the second-order offset coefficient;
*β_ij_*—the interaction coefficient;
*X_i_*, *X_j_*, and *X_i_X_j_*—the level values of each factor to analyze the main effect and interaction effect of each factor.

#### 3.2.4. Preparation of Nano HA-FeS

A stock solution of 1000 mg/L of HA was prepared using deionisation and stored at 4 °C protected from light. To the sterilized and cooled optimized medium, 1, 2, 5, 10, 20 and 50 mL of HA solution were added, resulting in a concentration of HA in the medium of 1, 2, 5, 10, 20 and 50 mg/L. The medium was placed in a thermostatic incubator (30 °C) for 7 days of static light incubation.

#### 3.2.5. Cr(VI) Removal Kinetic Experiments

To investigate the kinetics of the FeS–Cr(VI) reaction, 1 g of biologically synthesized nanoscale FeS and HA-stabilized nanoscale FeS were added to separate 100 mL aqueous samples, each with an initial Cr(VI) concentration of 100 mg/L at pH 4. The mixtures were incubated on a thermostatic shaker set to 30 °C and 150 r/min. During the first 60 min, samples were collected every 10 min, with additional samples taken at 90, 120, and 180 min to observe later-stage changes. All samples were immediately filtered through 0.22 μm membrane filters, and Cr(VI) concentrations were measured using the diphenylcarbazide spectrophotometric method (GB 7467-1987) [59].

Pseudo-first-order kinetic equation:

(3) $$q_{t}=q_{e}\left(1-e^{-kt}\right)\;$$

pseudo-second-order kinetic equation:

(4) $$\frac{t}{q_{t}}=\frac{1}{k_{2}q_{e}^{2}}+\frac{t}{q_{e}}$$

Intraparticle diffusion model:

(5) $$q_{t}=k_{p}t^{\frac{1}{2}}+C$$

*q_t_*—the adsorption capacity of the material for pollutants at time t, mg/g;
*q_e_*—Adsorption capacity of the material for the pollutant at equilibrium, mg/g;
*k*_1_—the pseudo-first-order adsorption rate constant, min^−1^;
*k*_2_—Pseudo-second-order adsorption rate constant, mg/(g·min);
*k_p_*—Intraparticle diffusion rate constant, mg/(g·min^1/2^);
*C*—Parameters related to the boundary layer, mg/g.

#### 3.2.6. Isothermal Adsorption Experiment

Isothermal adsorption experiments were conducted using water samples with a pH of 4 and initial Cr(VI) concentrations of 50, 100, 150, and 200 mg/L. In each conical flask, 1 g of optimized biological nano-FeS and HA-FeS was added to 100 mL of Cr(VI) solution at varying concentrations. The flasks were then placed on a constant-temperature shaker set to 30 °C and 150 r/min for oscillation. After 60 min, samples were filtered through a 0.22 μm microporous membrane, and the Cr(VI) concentration was immediately measured using the diphenylcarbazide spectrophotometric method (GB 7467-1987) [59].

Langmuir isothermal adsorption model:

(6) $$\frac{C_{e}}{q_{e}}=\frac{1}{q_{m}k_{L}}+\frac{C_{e}}{q_{m}}$$

Freundlich isothermal adsorption model:

(7) $$lnq_{e}=lnk_{F}+\frac{1}{n}lnC$$

*C_e_*—the concentration of Cr(VI) at solution equilibrium, mg/L;
*q_e_*—the material’s equilibrium adsorption capacity for pollutants, measured in mg/g;
*q_m_*—the material’s saturated adsorption capacity for pollutants, measured in mg/g;
*k_L_*—Adsorption constant of the Langmuir model;
*k_F_*—Adsorption constant of the Freundlich model;
*n*—Constant related to adsorption strength.

#### 3.2.7. Characterization

TEM (JEOL JEM-2100F, JEOL, Tokyo, Japan) was used to observe particle morphology after N_2_-protected drying on copper grids. SEM (FEI Inspect F50, FEI, Hillsboro, OR, USA) characterized surface morphology. FTIR (Thermo Fisher Nicolet iS5, KBr pellet, Thermo Fisher Scientific, Waltham, MA, USA) was collected from 400 to 4000 cm^−1^. XRD (Rigaku SmartLab 9, Rigaku, Tokyo, Japan) was performed on vacuum-dried solids (70 °C) before and after reaction to assess phase evolution. The Zeta potential of biological nano-FeS and HA-FeS was measured with a Malvern Zetasizer Nano ZS ZEN3600 Zeta potential analyzer (Malvern Panalytical, Almelo, The Netherlands) at pH levels of 4, 6, 7, 8, and 10 to quantify the changes in surface charge of materials.

## 4. Conclusions

This work demonstrates that HA stabilization can substantially enhance both the production and the reactivity of biogenic nano-FeS for remediating acidic Cr(VI)-bearing wastewater, a matrix in which Cr(VI) typically remains highly soluble and difficult to immobilize. Optimization via single-factor tests and response surface methodology identified a yeast extract powder dosage of 2.2 g/L, an Fe/S molar ratio of 0.8, and an NH_4_Cl dosage of 3.1 g/L as the most favorable preparation conditions, yielding 84.25% Cr(VI) removal and confirming that the Fe/S ratio exerts the strongest control on FeS formation and performance. Introducing HA at an optimal dosage of 2 mg/L further shifted the system to near-complete Cr(VI) removal (99.62%), lowering residual Cr(VI) from 15.75 mg/L to 0.38 mg/L and simultaneously increasing the nano-FeS yield to 1096.26 mg/L (an increment of 385.51 mg/L relative to the HA-free system).

Kinetic and isotherm analyses support a surface-controlled Cr(VI) removal process with heterogeneous binding sites, matching microscopy and electrokinetic observations of improved dispersion, reduced lamellar stacking, and a higher absolute zeta potential after HA stabilization. FTIR coupled with 2D-COS further points to coordinated changes in O–H/N–H (~3294 cm^−1^), C=O (~1640 cm^−1^), and C–O (~1040 cm^−1^) vibrations, implicating oxygen-containing, HA-derived functionalities in interfacial interactions during Cr(VI) removal. XRD provides direct evidence that Cr(VI) is reduced to Cr(III) and predominantly immobilized as CrOOH and Cr_2_S_3_; by contrast, any Fe–Cr spinel-like phase assignment should be regarded as tentative because XPS confirmation is not available.

Future investigations utilizing surface-sensitive spectroscopy (XPS) together with long-term leaching tests under dynamic geochemical conditions representative of AMD are needed to constrain Cr(VI) speciation more rigorously and to evaluate the durability of the immobilized products. Overall, this study provides a green, efficient, and cost-effective strategy for Cr(VI) remediation in AMD, addressing the core stability challenges of biogenic FeS nanoparticles and offering valuable insights for the development of high-performance remediation materials for AMD.

## Figures and Tables

**Table 1:** The experimental results of RSM.

No.	Variable						
	Actual Value			Encoded Value			Cr(VI) Response
	A	B	C	A	B	C	/%
1	2.00	0.90	2.00	0	1	−1	81.41
2	2.00	0.50	3.00	0	0	0	83.46
3	1.00	0.90	3.00	−1	1	0	80.2
4	2.00	0.10	4.00	0	−1	1	65.42
5	2.00	0.50	3.00	0	0	0	83.22
6	2.00	0.10	2.00	0	−1	−1	63.17
7	2.00	0.50	3.00	0	0	0	82.78
8	3.00	0.50	4.00	1	0	1	78.29
9	3.00	0.90	3.00	1	1	0	82.59
10	1.00	0.10	3.00	−1	−1	0	60.1
11	2.00	0.50	3.00	0	0	0	83.44
12	2.00	0.90	4.00	0	1	1	82.46
13	1.00	0.50	2.00	−1	0	−1	70.54
14	3.00	0.50	2.00	1	0	−1	76.83
15	3.00	0.10	3.00	1	−1	0	70.78
16	2.00	0.50	3.00	0	0	0	83
17	1.00	0.50	4.00	−1	0	1	73.54

**Table 2:** Analysis of Variance of Regression model for Cr(VI) Removal Rate.

Source	Sum of Square	Degrees of Freedom	Mean Square	F	*p*	Salience
Model	981.12	9	109.01	312.37	<0.0001	significant
A	72.66	1	72.66	208.21	<0.0001	***
B	564.31	1	564.31	1616.99	<0.0001	***
C	7.53	1	7.53	21.57	0.0024	**
AB	17.18	1	17.18	49.23	0.0002	***
AC	0.59	1	0.59	1.70	0.2337	⊙
BC	0.36	1	0.36	1.03	0.3436	⊙
A^2^	68.68	1	68.68	196.80	<0.0001	***
B^2^	137.94	1	137.94	395.26	<0.0001	***
C^2^	79.35	1	79.35	227.38	<0.0001	***
Residual tern	2.44	7	0.35			
Lack of fit	2.10	3	0.70	8.25	0.0346	
Pure error	0.34	4	0.085			
Sum	983.56	16				
Coefficient of variation	0.77%	Adeq Precision	51.309	R^2^ = 0.9975	R^2^_Adj_ = 0.9943	R^2^_Pred_ = 0.9653

Note: ***, *p* < 0.001, extremely significant; **, *p* < 0.01, highly significant; ⊙, not significant.

**Table 3:** Optimization results for biogenic FeS nanoparticle preparation using response surface methodology.

Parameter	A	B	C	Cr(VI) Removal Rate
Predicted	2.19 g/L	0.78	3.08 g/L	86.43%
Experimental	2.2 g/L	0.8	3.1 g/L	84.25%

**Table 4:** Kinetic parameters for the removal of Cr(VI) by biological nano-FeS and HA-FeS.

Kinetic Model	Parameters	Biogenic Nano-FeS	HA-FeS
Pseudo-first-order model	*K*_1_	0.0709	0.1007
	R^2^	0.9231	0.7782
	*q_e_*	308.0586	353.9556
Pseudo-second-order model	*K*_2_	0.0004	0.0005
	R^2^	0.9985	0.9991
	*q_e_*	328.9474	378.7879
Intraparticle diffusion model	*K_p_*_1_	31.4508	27.1774
	R^2^	0.9985	0.9997
	*K_p_*_2_	__	22.5719
	R^2^	__	0.9548
	*K_p_*_3_	1.5145	0.2109
	R^2^	0.85304	0.38776

**Table 5:** Fitting parameters of adsorption isotherms of Cr(VI) on biological nano-FeS and HA-FeS.

	Parameters	Biogenic Nano-FeS	HA-FeS
Langmuir isothermal adsorption model:	*K_L_*	0.0680	0.3968
	*q_m_*	621.1180	574.7126
	R^2^	0.9385	0.9904
Freundlich isothermal adsorption model:	*k_F_*	59.6862	152.5801
	*n*	1.9465	2.9678
	R^2^	0.8581	0.9400

**Table 6:** Zeta potentials of biological nano-FeS and HA-FeS.

pH	Zeta (mv)	
	FeS	HA-FeS
4	−8.32	3.22
6	−12.33	−16.47
7	−16.87	−21.3
8	−20.2	−24.43
10	−28.67	−30.4

**Table 7:** RSM design factor horizontal coding table.

Factor	Code		Coding Level	
		−1	0	1
Yeast extract powder /(g/L)	A	1	2	3
Fe/S molar ratio	B	0.1	0.5	0.9
NH_4_Cl dosage /(g/L)	C	2	3	4

## Data Availability

The data presented in this study are available on request from the corresponding author.

## Supplementary Materials

The following supporting information can be downloaded at: https://www.mdpi.com/article/10.3390/molecules31060962/s1, Figure S1: The SEM and TEM images illustrate the nano-FeS synthesized using varying amounts of yeast extract powder; Figure S2: SEM and TEM images depict nano-FeS synthesized at varying Fe/S molar ratios; Figure S3: SEM and TEM images of nano-FeS prepared with different NH_4_Cl dosage; Figure S4: SEM and TEM images of nano FeS and HA-FeS before and after reaction. (a-1) SEM image of bio-logical nano-FeS before the reaction; (b-1) SEM image of biological nano-FeS after the reaction; (c-1) SEM image of HA-FeS before the reaction; (d-1) SEM image of HA-FeS after the reaction; (a-2) TEM image of biological nano-FeS before the reaction; (b-2) TEM image of biological nano-FeS after the reaction; (c-2) TEM image of HA-FeS before the reaction; (d-2) TEM image of HA-FeS after the reaction.

## References

[1] Sun J., Luo Y., Ye J., Li C., Shi J. (2022). Chromium Distribution, Leachability and Speciation in a Chrome Plating Site. Processes.

[2] Zulfiqar U., Haider F.U., Ahmad M., Hussain S., Maqsood M.F., Ishfaq M., Shahzad B., Waqas M.M., Ali B., Tayyab M.N. (2023). Chromium toxicity, speciation, and remediation strategies in soil-plant interface: A critical review. Front. Plant Sci..

[3] Dhal B., Thatoi H., Das N., Pandey B. (2013). Chemical and microbial remediation of hexavalent chromium from contaminated soil and mining/metallurgical solid waste: A review. J. Hazard. Mater..

[4] Tumolo M., Ancona V., De Paola D., Losacco D., Campanale C., Massarelli C., Uricchio V.F. (2020). Chromium Pollution in European Water, Sources, Health Risk, and Remediation Strategies: An Overview. Int. J. Environ. Res. Public Health.

[5] Islam M., Mohana A.A., Rahman A., Rahman M., Naidu R., Rahman M.M. (2023). A Comprehensive Review of the Current Progress of Chromium Removal Methods from Aqueous Solution. Toxics.

[6] RoyChowdhury A., Sarkar D., Deng Y., Datta R. (2016). Assessment of Soil and Water Contamination at the Tab-Simco Coal Mine: A Case Study. Mine Water Environ..

[7] Chen M., Lu G., Guo C., Yang C., Wu J., Huang W., Yee N., Dang Z. (2015). Sulfate migration in a river affected by acid mine drainage from the Dabaoshan mining area, South China. Chemosphere.

[8] Liu S., Gao H., Cheng R., Wang Y., Ma X., Peng C., Xie Z. (2020). Study on influencing factors and mechanism of removal of Cr(VI) from soil suspended liquid by bentonite-supported nanoscale zero-valent iron. Sci. Rep..

[9] Feng X.-J., Wang X.-Y., Li D.-M., Liu Z.-H., Yan Y.-L. (2023). Sulfidation of nano zero-valent iron for enhanced hexavalent chromium removal performance. Water Sci. Eng..

[10] Yu J., Yang S., Liu D., Yang Z., Xu J., Li Y., Tang Z. (2023). Interaction of chromium-sulfur-iron during Cr(VI) stabilization by polysulfide-modified nanoscale zero-valent iron for groundwater remediation: Batch experiments and numerical simulation. Chem. Eng. J..

[11] Tan X., Shaaban M., Yang J., Cai Y., Wang B., Peng Q.-A. (2021). Efficient Removal of Hexavalent Chromium from an Aquatic System Using Nanoscale Zero-Valent Iron Supported by Ramie Biochar. Nanomaterials.

[12] Jeyakumar P., Debnath C., Vijayaraghavan R., Muthuraj M. (2022). Trends in Bioremediation of Heavy Metal Contaminations. Environ. Eng. Res..

[13] Syawaluddin L.O.M., Retnaningrum E. (2022). Chromium bioremediation of batik industrial wastewater using a consortium of sulfate-reducing bacteria from forested wetland soil. J. Degraded Min. Lands Manag..

[14] Guo Y., Cao D., Tang S., Hu Y., Dong W., Wu X. (2025). Efficient Chromium(VI) Removal Through In Situ Nano-Iron Sulfide Formation at the Cathode of Microbial Fuel Cells. Water.

[15] Fang S., Huang X., Xie S., Du J., Zhu J., Wang K., Zhuang Q., Huang X. (2022). Removal of Chromium (VI) by a Magnetic Nanoscale Zerovalent Iron–Assisted Chicken Manure-Derived Biochar: Adsorption Behavior and Synergetic Mechanism. Front. Bioeng. Biotechnol..

[16] Li Q., Liu M., Qiu X., Liu X., Dapaah M.F., Niu Q., Cheng L. (2022). Removal of Chromium(VI) by Nanoscale Zero-Valent Iron Supported on Melamine Carbon Foam. Nanomaterials.

[17] Salama E., Samy M., Shokry H., El-Subruiti G., El-Sharkawy A., Hamad H., Elkady M. (2022). The superior performance of silica gel supported nano zero-valent iron for simultaneous removal of Cr (VI). Sci. Rep..

[18] Sun Y., Liu J., Fan X., Li Y., Peng W. (2023). Synthesis and application of iron sulfide−based materials to activate persulfates for wastewater remediation: A review. Front. Environ. Sci..

[19] Sun Y., Zheng F., Wang W., Zhang S., Wang F. (2020). Remediation of Cr(VI)-Contaminated Soil by Nano-Zero-Valent Iron in Combination with Biochar or Humic Acid and the Consequences for Plant Performance. Toxics.

[20] Zykova M.V., Volikov A.B., Buyko E.E., Bratishko K.A., Ivanov V.V., Konstantinov A.I., Logvinova L.A., Mihalyov D.A., Sobolev N.A., Zhirkova A.M. (2023). Enhanced Antioxidant Activity and Reduced Cytotoxicity of Silver Nanoparticles Stabilized by Different Humic Materials. Polymers.

[21] Joseph S., Cowie A.L., Van Zwieten L., Bolan N., Budai A., Buss W., Cayuela M.L., Graber E.R., Ippolito J.A., Kuzyakov Y. (2021). How biochar works, and when it doesn’t: A review of mechanisms controlling soil and plant responses to biochar. GCB Bioenergy.

[22] Wyszkowska J., Borowik A., Zaborowska M., Kucharski J. (2022). Sensitivity of *Zea mays* and Soil Microorganisms to the Toxic Effect of Chromium (VI). Int. J. Mol. Sci..

[23] Wang X., Muhmood A., Yu H., Li Y., Fan W., Tian P. (2022). Unveiling the Potential of Novel Struvite–Humic Acid Composite Extracted from Anaerobic Digestate for Adsorption and Reduction of Chromium. Catalysts.

[24] Coelho F.E.B., Candelario V.M., Araújo E.M.R., Miranda T.L.S., Magnacca G. (2020). Photocatalytic Reduction of Cr(VI) in the Presence of Humic Acid Using Immobilized Ce–ZrO_2_ under Visible Light. Nanomaterials.

[25] Ka-Ot A.L., Joshi S.R. (2021). Application of acid and heavy metal resistant bacteria from rat-hole coal mines in bioremediation strategy. J. Basic Microbiol..

[26] Tang J.L., He H., Zhang W.J., Hong F.F., Zhang S.Y., Sun M., Tao X.X. (2014). Isolation and identification of SRB and its utilization on processing of acid mine drainage of coal gangue dump. J. China Coal Soc..

[27] Cheng J., Zuo J.W. (2010). Improvement of bioavailability of cobalt and iron by yeast extract in anaerobic system. China Environ. Sci..

[28] Zhang K., Liu H.C., Li J.Z. (2006). Effect of ecological factors on sulfate removal rate in acidogenic sulfate-reducing reactor. J. N. China Univ. Water Resour. Electr. Power (Nat. Sci. Ed.).

[29] Wang J.W. (2020). The Change of Mechanism in FeS Remediation of Heavy Metal under Oxidizing Environments and the Regeneration of FeS Reactivity by Sulfate Reducing Bacteria. Master’s Thesis.

[30] Gu F.Y. (2017). The Study on Nitrogen Source of Sulfate Reducing Bacteria Culture Medium and the Treatment Conditions of Acid Mine Drainage. Master’s Thesis.

[31] Di J., Wang M., Zhu Z. (2018). Experiment on the treatment of acid mine drainage with optimized biomedical stone particles by response surface methodology. Environ. Sci. Pollut. Res..

[32] Ayilara M.S., Babalola O.O. (2023). Bioremediation of environmental wastes: The role of microorganisms. Front. Agron..

[33] Zhang J., Mostofa K.M.G., Yang X., Mohinuzzaman M., Liu C.-Q., Senesi N., Senesi G.S., Sparks D.L., Teng H.H., Li L. (2023). Isolation of dissolved organic matter from aqueous solution by precipitation with FeCl_3_: Mechanisms and significance in environmental perspectives. Sci. Rep..

[34] Xu Z., Tsang D.C. (2022). Redox-induced transformation of potentially toxic elements with organic carbon in soil. Carbon Res..

[35] Huang Y., Tang J., Zhang B., Long Z.-E., Ni H., Fu X., Zou L. (2023). Influencing factors and mechanism of Cr(VI) reduction by facultative anaerobic Exiguobacterium sp. PY14. Front. Microbiol..

[36] Hou Y., Li Y., Wang Y., Zhu Z., Tang S., Zhang J., Pan Q., Hu T. (2024). Goethite Enhances Cr(VI) Reduction by *S. oneidensis* MR-1 under Different Conditions: Mechanistic Insights. Microorganisms.

[37] Chang J., Zhang J., Wang H., Bai Y., Liu Y., Bi Y., Zhang H., Chen H., Barnie S., Xie H. (2022). Cr(VI) adsorption and reduction by magnetite-humic acid adsorption complexes under mildly acidic conditions: Synergistic/antagonistic mechanism and multi-step reaction model. Chem. Eng. J..

[38] Lin Z., Zheng C., Wang Z., Peng Y., Geng G., Zhu A., He C. (2024). Rationally engineered core-shell structured Fe0@FeS towards the efficacious synergistic Cr(VI) and Ni(II) removal. Chem. Eng. J..

[39] Morales A., Ratcliff J., Hodonj D., Lott P., Deutschmann O., Bollini P., Harold M. (2025). Elucidating Rate-Determining Steps of Surface-Catalyzed Reactions Exhibiting Isothermal Rate Multiplicity. ACS Catal..

[40] Liu S., Wang M., Yang X., Shi Q., Qiao Z., Lucero M., Ma Q., More K.L., Cullen D.A., Feng Z. (2020). Chemical Vapor Deposition for Atomically Dispersed and Nitrogen Coordinated Single Metal Site Catalysts. Angew. Chem. Int. Ed. Engl..

[41] Zheng S., Wang X., Liu L., Yuan X., Zhu Z. (2025). Study on isothermal kinetics of CO reduction roasting nickel laterite ore in micro-fluidized bed. Chem. Eng. J..

[42] Maftei A.E., Lupu A., Rodriguez-Blanco J.D., Rateau R., Brinza L. (2025). Chromium removal via coprecipitation with carbonates and iron oxyhydroxides minerals: The effect of organic complexing agents. Sci. Total. Environ..

[43] Deshmukh A., Elimelech M., Lienhard J.H. (2025). Partition–diffusion–reaction bounds for thin-film membrane formation kinetics. Chem. Eng. J..

[44] Yao Y., Mi N., He C., He H., Zhang Y., Zhang Y., Yin L., Li J., Yang S., Li S. (2020). Humic acid modified nano-ferrous sulfide enhances the removal efficiency of Cr(VI). Sep. Purif. Technol..

[45] Fenti A., Chianese S., Iovino P., Musmarra D., Salvestrini S. (2020). Cr(VI) Sorption from Aqueous Solution: A Review. Appl. Sci..

[46] Luo C., Xiong C., Hou Y., Qiu Y. (2025). Ligand-selective complexation of natural organic matter with Mg^2+^ modulates nanoplastic transport in seawater-saturated porous media. Water Res..

[47] Xiao Y., Li Y., Yang H., Rashid S., Graham N., Yu W. (2025). The irreversible transformation of the molecular structure of humic acid during pH change and its effects on the formation of disinfection by-products. J. Hazard. Mater..

[48] Wang T., Zhao D., Liu J., Zhang T., Wang X., Liu T., Wang S., Liu G., Liu B., Liu Y. (2023). Effects of abiotic mineral transformation of FeS on the dynamic immobilization of Cr(VI) in oxic aquatic environments. Sci. Total. Environ..

[49] Shi S., Zhang C., Guo S., Yang L., Pan Y., Zhou M., Zhang Y. (2025). Surface hydroxyl-driven =FeOFe(II)^+^ and =FeOH_2_^+^ generation on polyphenol-modified ZVI: Mechanistic insights into Cr(VI) and surface passivation layer removal. J. Hazard. Mater..

[50] Tao C., Wu K., Liu T., Yang S., Li Z. (2025). One-step removal of p-arsanilic acid via constructing bifunctional Zr-CuO/α-FeOOH catalyst: Efficient peroxymonosulfate activation, cooperative oxidation and adsorption. Chem. Eng. J..

[51] Wang Q., Xu Q., Liu W., Jiao M., Chen Z., Wang A. (2024). Transforming contaminant ligands at water–solid interfaces via trivalent metal coordination. Environ. Int..

[52] Guo X., Zhang H., Li Z., Lin C., Zhang J., Wang Y., Pang Y. (2025). Immobilization mechanisms of Cr(VI)/Cr(III) in red mud-based binder: Insights from Cr-S-Fe interactions and hydration gel structure evolution. J. Hazard. Mater..

[53] Zhou J., Yin Y., Chen J., Deng Y., Sun R., Song C., Wei Z. (2025). Toward sulfur retention and H _2_ S mitigation in composting: Elucidating and validating the molecular pathway of Fe(ii)/Fe(iii)-driven sulfur incorporation into humic acid. Green Chem..

[54] Qin X., Desmau M., Guinoiseau D., Rovezzi M., Gélabert A., Ren Z., Benedetti M.F. (2025). Unveiling chromium dynamics: XANES spectroscopy insights in two Fe-Mn nodule-rich red soil profiles. J. Hazard. Mater..

[55] Tang H., Chen M., Wu P., Li Y., Wang T., Wu J., Sun L., Shang Z. (2024). The influence of Mn(II) on transformation of Cr-absorbed Schwertmannite: Mineral phase transition and elemental fate. Water Res..

[56] Guo Q., Yu D., Yang J., Zhao T., Yu D., Li L., Wang D. (2024). A novel sequential extraction method for the measurement of Cr(VI) and Cr(III) species distribution in soil: New insights into the chromium speciation. J. Hazard. Mater..

[57] Wang W., Fang X., Fu Q.-L., Huang C., Fang L., Zhang J., Feng X., Tan W., Liu F., Li J. (2025). Iron(II/III) Alters the Relative Roles of the Microbial Byproduct and Humic Acid during Chromium(VI) Reduction and Fixation by Soil-Dissolved Organic Matter. Environ. Sci. Technol..

[58] Shi Y., Sheng A., Wu X., Qi W., Crittenden J.C., Chen J., Wang L. (2025). Enhanced performance of calcium alginate-modified ferrous sulfide on the adsorption of Pb, Zn, and Cd: Mechanism and stability of the products. Chem. Eng. J..

[59] (1987). Water Quality—Determination of Chromium(VI)—1,5-Diphenylcarbohydrazide Spectrophotometric Method.

