# Supplementary material for: Humic Acid-Stabilized Biogenic FeS Nanoparticles for Cr(VI) Removal Under Simulated Acidic Mine Drainage Conditions: Optimization and Interfacial Transformation Pathways
Source: Molecules. 2026 Mar 12;31(6):962. doi: 10.3390/molecules31060962 (PMC13029606; doi:10.3390/molecules31060962)
Supplement: Supplementary file 1 [file molecules-31-00962-s001.zip › molecules-4148073-supplementary.pdf]

Supplementary Materials

Article

Humic Acid-Stabilized Biogenic FeS nanoparticles for Cr(VI) Removal Under Simulated Acidic Mine Drainage Conditions: Optimization and Interfacial Transformation Pathways

Mengjia Dai [1], Junzhen Di [2,*] and Min Zhang [3]

[1] College of Mining, Liaoning Technical University, Fuxin 123000, China; 18342812950@163.com
[2] College of Civil Engineering, Liaoning Technical University, Fuxin 123000, China
[3] College of Environmental Science and Engineering, Liaoning Technical University, Fuxin 123000, China; 19824856906@163.com
* Correspondence: dijunzhen@126.com; Tel.: +86-139-4188-9524

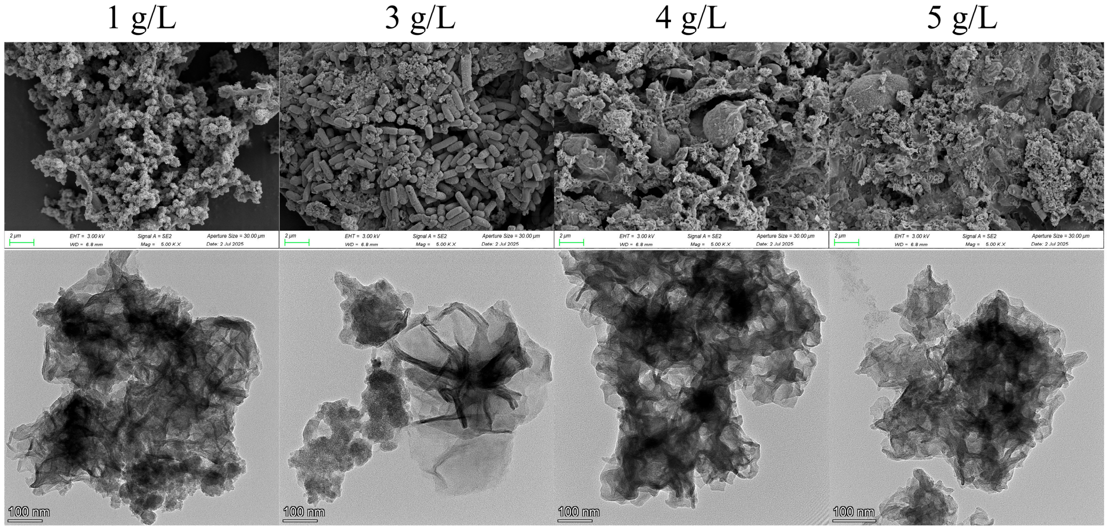

Figure S1. The SEM and TEM images illustrate the nano-FeS synthesized using varying amounts of yeast ex-tract powder (Corresponding to Figure 1b in the manuscript)

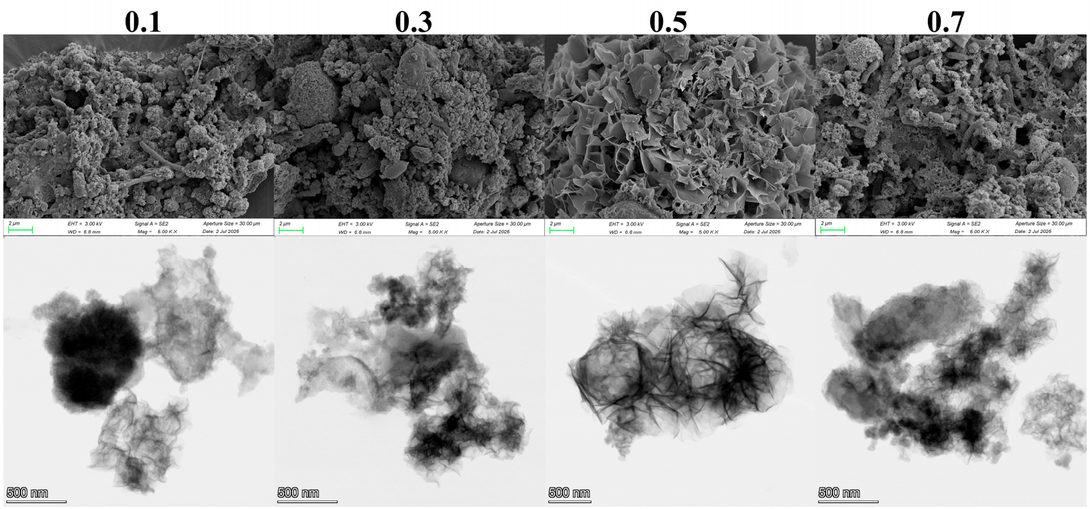

Figure S2. SEM and TEM images depict nano-FeS synthesized at varying Fe/S molar ratios (Corresponding to Figure 2b in the manuscript)

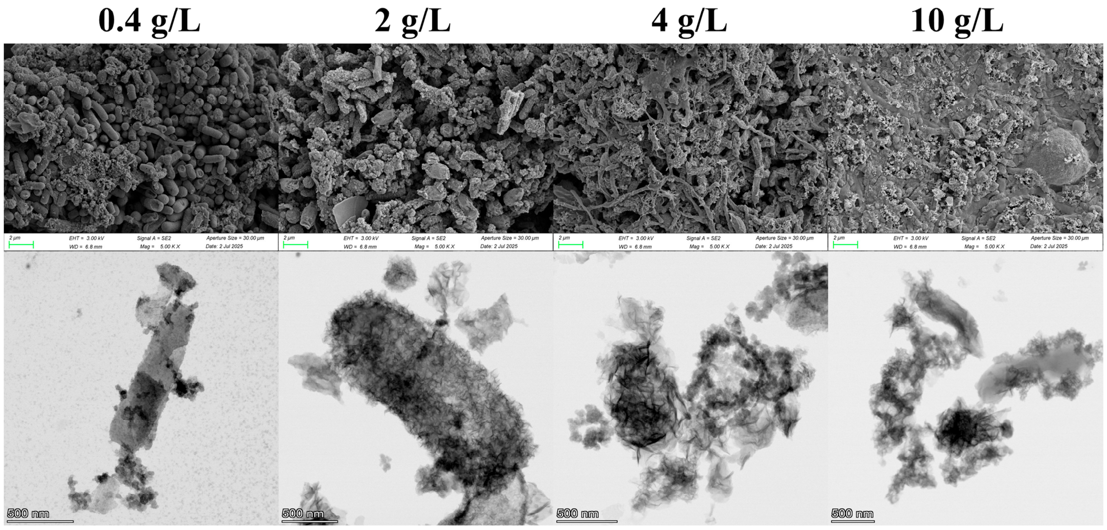

Figure S3. SEM and TEM images of nano-FeS prepared with different NH_4Cl dosage (Corresponding to Figure 3b in the manuscript)

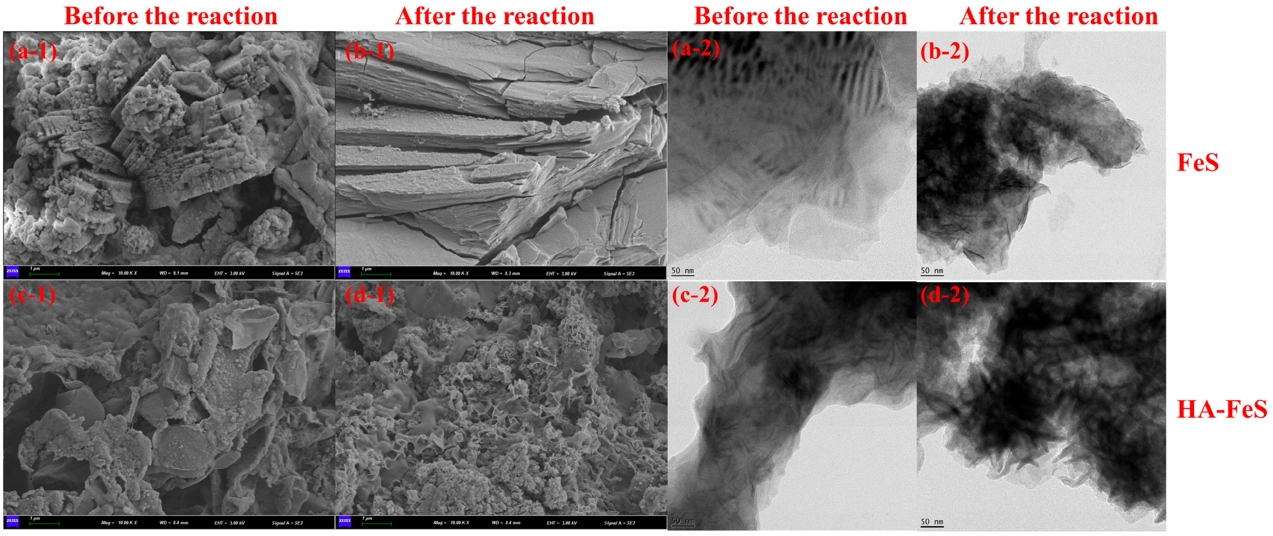

Figure S4. SEM and TEM images of nano FeS and HA-FeS before and after reaction. (a-1) SEM image of bio-logical nano-FeS before the reaction; (b-1) SEM image of biological nano-FeS after the reaction; (c-1) SEM image of HA-FeS before the reaction; (d-1) SEM image of HA-FeS after the reaction; (a-2) TEM image of biological nano-FeS before the reaction; (b-2) TEM image of biological nano-FeS after the reaction; (c-2) TEM image of HA-FeS before the reaction; (d-2) TEM image of HA-FeS after the re-actio (Corresponding to Figure 8 in the manuscript)
